# Development of a selection assay for small guide RNAs that drive efficient site-directed RNA editing

**DOI:** 10.1093/nar/gkad098

**Published:** 2023-02-25

**Authors:** Juan Felipe Diaz Quiroz, Namrata Ojha, Elnur E Shayhidin, Dasuni De Silva, Jesse Dabney, Amy Lancaster, James Coull, Stuart Milstein, Andrew W Fraley, Christopher R Brown, Joshua J C Rosenthal

**Affiliations:** Eugene Bell Center for Regenerative Biology and Tissue Engineering, The Marine Biological Laboratory, Woods Hole, MA, USA; Eugene Bell Center for Regenerative Biology and Tissue Engineering, The Marine Biological Laboratory, Woods Hole, MA, USA; Korro Bio Inc., Cambridge, MA, USA; Korro Bio Inc., Cambridge, MA, USA; Korro Bio Inc., Cambridge, MA, USA; Korro Bio Inc., Cambridge, MA, USA; Korro Bio Inc., Cambridge, MA, USA; Korro Bio Inc., Cambridge, MA, USA; Korro Bio Inc., Cambridge, MA, USA; Korro Bio Inc., Cambridge, MA, USA; Eugene Bell Center for Regenerative Biology and Tissue Engineering, The Marine Biological Laboratory, Woods Hole, MA, USA

## Abstract

A major challenge confronting the clinical application of site-directed RNA editing (SDRE) is the design of small guide RNAs (gRNAs) that can drive efficient editing. Although many gRNA designs have effectively recruited endogenous Adenosine Deaminases that Act on RNA (ADARs), most of them exceed the size of currently FDA-approved antisense oligos. We developed an unbiased *in vitro* selection assay to identify short gRNAs that promote superior RNA editing of a premature termination codon. The selection assay relies on hairpin substrates in which the target sequence is linked to partially randomized gRNAs in the same molecule, so that gRNA sequences that promote editing can be identified by sequencing. These RNA substrates were incubated *in vitro* with ADAR2 and the edited products were selected using amplification refractory mutation system PCR and used to regenerate the substrates for a new round of selection. After nine repetitions, hairpins which drove superior editing were identified. When gRNAs of these hairpins were delivered *in trans*, eight of the top ten short gRNAs drove superior editing both *in vitro* and *in cellula*. These results show that efficient small gRNAs can be selected using our approach, an important advancement for the clinical application of SDRE.

## INTRODUCTION

Over the last two decades, a new area of precision medicine has emerged that utilizes strategically designed oligonucleotides which hybridize to a target RNA and recruit endogenous enzymatic activities for the treatment of disease. For example, synthetic oligonucleotides have been used to modulate RNA splicing (i.e. splice switching) (1) or reduce mRNA expression levels by nucleating RNAi pathways (2) along with a host of other applications (3). In addition, chemically stabilized exogenous mRNA has been used to recruit ribosomes to produce proteins for vaccines and protein-replacement therapies (4,5). Recently, the recruitment of Adenosine Deaminases that Act on RNAs (ADARs) to direct site-specific editing of RNA has garnered interest as a potential therapeutic modality (6). ADARs catalyze the conversion of Adenosine (A) to Inosine (I), a biological mimic for Guanosine (G) during translation and other cellular processes. Base recoding by ADARs can be used to correct G-to-A mutations, modulate protein function, or regulate protein splicing and expression when directed to introns within pre-mRNAs or untranslated regions (UTRs) in mature mRNAs. Humans, like all vertebrates, express two catalytically active ADAR enzymes (ADAR1 and ADAR2) (7). ADARs, because they are active across numerous tissues, make ideal targets for recruitment by antisense oligonucleotides (ASOs).

The general goal of therapeutic RNA editing is to recruit endogenous ADAR proteins and induce them to edit a specific adenosine by creating an editable structure using an ASO. Natural ADAR substrates are normally large, higher order hairpin-like structures within mRNAs composed of imperfect duplexes, mismatches, and bulges (8–15). Often, these structures are formed between intronic and exonic sequences in pre-mRNAs with required structural elements lying within the same molecule. In contrast, structures that drive therapeutic editing are created by an ASO delivered *in trans*. ASO design can vary tremendously, and many can promote efficient editing in both cultured cells and animal models. For example, some ASOs contain domains that mimic naturally occurring ADAR substrates (16,17) while other ASOs are relatively long, creating near perfect duplexes with the substrate (18). Recently, it has been reported that circular RNAs can efficiently recruit ADAR activity (19,20). While these designs can be effective, they suffer from a significant drawback of being relatively large, ranging in size from 75 to 200 nucleotides (nt). On the other hand, to date, therapeutic ASOs used for applications outside of ADAR editing are far smaller. Currently, there are 15 FDA approved ASOs for therapeutics, and their sizes range from 18 to 30 nt (21,22). In fact, there has been only one report of successful ADAR recruitment using an ASO in this size range (in this case a 30mer) (23). Thus, the design of short oligos that can successfully recruit endogenous ADAR remains a major challenge in the field.

Examples of small, natural structures from yeast and human mRNAs and their derivatives that recruit ADAR exist and are similar in size to structures formed by 20–30 nt ASOs *in trans* (24,25). In addition, these smaller structures drive RNA editing at rates among the fastest reported. The challenge lies in the fact that no universal rules exist on how to design small structures to drive editing. Although it is clear that perfect RNA duplexes make suboptimal structures for editing (26), the positions and chemical identities of mismatches and bulges are difficult to predict. Accordingly, unbiased screening assays can be used to identify the best performing ASOs out of randomized pools.

High-throughput screens are a mainstay in the pharmaceutical industry for drug discovery (27,28). When used to identify ASOs for ADAR recruitment, the design of such screens is critical because the mutational space is exceptionally large. For example, a short 20mer has 10^12^ distinct variations, based on sequence alone. For a 30mer there are ∼10^18^ variations. Although screening this many molecules is beyond the capabilities of cell-based systems in standard research labs, for any approach, the more molecules that can be interrogated, the more powerful the screen. Because of its high transformation potential (up to 10^10^ colonies/μg plasmid) *E. coli* would be an ideal host for a screening assay. Unfortunately, ADAR cannot be expressed in bacteria because it requires inositol hexakisphosphate as a cofactor for expression (29). Accordingly, ADARs are typically expressed in eukaryotic cells. With standard transformation rates for yeast at ∼10^6^ and for cultured cells at ∼10^4^, the use of these systems allows only a tiny fraction of the mutational space to be interrogated. By contrast, *in vitro* (i.e. cell-free) screening, allows for the interrogation of ∼10^10^ molecules. Although still below the theoretical mutational space, *in vitro* screening allows for evaluation of orders of magnitude more molecules than *in cellula* (i.e. cultured cells) based approaches.

The fact that therapeutic ASOs and target mRNAs form double-stranded RNA (dsRNA) structures *in trans* presents another major logistical challenge for high-throughput screens. In this configuration, individual ASOs cannot be linked to editing outcomes because the ASO and the target are on different molecules. Linking the ASO and target together on the same construct (*in cis*), would enable identification of the ASOs that promote editing via sequencing. Deep sequencing platforms can yield up to ∼10^9^ reads, however this is still significantly below the number of ASOs needed to be screened. In addition, the fact that an ASO promotes editing once under favorable conditions (e.g. high ADAR concentration), does not make it a good candidate for promoting efficient editing under more stringent conditions. Further refinements to an *in vitro* approach are therefore required.

To increase the power of the *in vitro* platform for the selection of ADAR-recruiting ASOs, we have developed a strategy based on repetitive selection. ASOs with randomized mismatches are coupled to their target sequence *in cis* which is edited *in vitro* by ADAR. After each round of editing, edited targets are selected by Amplification Refractory Mutation System (ARMS) PCR (30,31) and this pool of molecules, including the ASOs that drove editing, is then prepared for the next round of editing. Thus, in each round of the assay, ASOs that promote editing are selected, and the ones that do not are eliminated, thereby reducing the mutational space to be screened and increasing the probability of identifying ASOs that promote superior editing. In this paper, we describe the development of this selection assay and its efficacy in identifying optimized, small guide RNAs (gRNAs) for ADAR-mediated A-to-I editing on mRNA. Using the selection assay, we identified 21mer and 31mer hairpins (*in cis*) that improved catalytic rates of editing *in vitro* when compared to a perfectly duplexed control. When the corresponding ASOs from these selected hairpins were generated and tested *in trans* (*in vitro* or *in cellula*), the 31mer ASOs, but not the 21mer ASOs, showed superior performance over the control ASOs.

## MATERIALS AND METHODS

### DNA construct assembly

**Figure 1.**
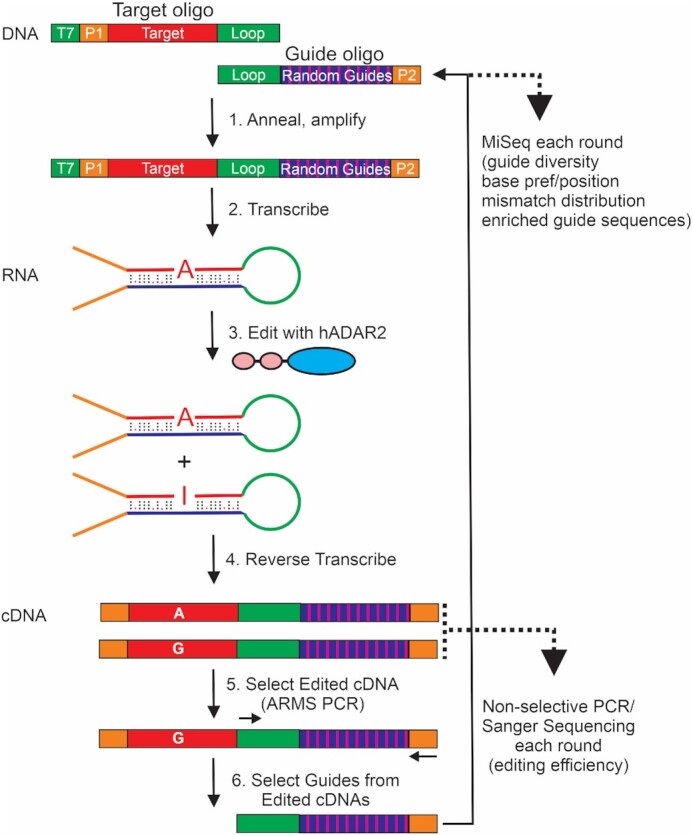
Selection assay. A DNA substrate was assembled by annealing the Target oligo containing a T7 promoter (green), a universal forward primer (P1, orange), the target sequence (red) and the connecting loop (green); and the Guide antisense oligo containing the connecting loop sequence (green), the randomized guides pool (striped purple) and a universal reverse primer (P2, orange). After annealing and amplification (Step 1), the DNA substrate was transcribed (Step 2) into RNA hairpins. The hairpins were incubated with HEK293T cell extracts overexpressing human ADAR2 (Step 3) leading to two possible outcomes, unedited or edited hairpins. The products of the editing reaction were reverse transcribed (Step 4) to obtain cDNA. The cDNA was then used as substrate for a Non-Selective PCR to monitor editing efficiency by Sanger sequencing (lower dotted arrow). The cDNA was also used as a substrate for a Selective PCR (ARMS PCR) (Step 5) to amplify only the substrates that were edited. From the products of the Selective PCR, the guide regions of the edited cDNAs were amplified (Step 6) and used (Guide oligo) to regenerate the substrate and start a new cycle of the assay. The regenerated substrate was also sent for MiSeq sequencing to monitor the diversity of the guide region (upper dotted arrow).

The DNA constructs to be used as templates for RNA transcription were synthesized by annealing and amplifying the target oligos and the guide oligos (Figure 1, Supplementary Table S1). The annealing/amplification PCR [0.5 μM target oligo, 1.5 μM guide oligo, 1 mM dNTPs in 20 μl total volume] was set up using Vent (exo-) polymerase [0.04 U/μl] (NEB). Because this polymerase lacks 3’ to 5’ and 5’ to 3’ exonuclease activity, the mismatches caused by the randomness in the guide will not be corrected by the polymerase. PCR annealing was set at 55°C for 30 s and run for 30 cycles. Afterwards, PCR products were run on a 2% agarose gel and purified.

The purified products were used as templates for another round of PCR amplification to add the sequence for the P2 primer and obtain the amount needed for RNA transcription. A 2-step PCR reaction [0.8 ng template, 375 nM forward primer, 375 nM reverse primer, 50 μM dNTPs in 40 μl total volume] was set up using the forward synthesis and P2 primers (Supplementary Table S1) and Vent(exo-) polymerase [0.02 U/μl]. The PCR products were column purified and the correct assembly was confirmed by agarose gel electrophoresis and Sanger sequencing (Azenta Life Sciences).

### RNA substrate generation

For each template, RNA was synthesized using the T7-FlashScribe Transcription Kit from CellScript. Up to 300 ng of template DNA was incubated at 37°C for 2 h in a 20 μl reaction volume. Afterwards, 2 μl of DNase I were added to the reaction and incubated at 37°C for an extra hour. To ensure the complete elimination of DNA, an extra DNase treatment was set up by adding 20 μl of 10× DNase I Buffer and 4 μl of DNase I enzyme (NEB), bringing the reaction volume to 200 μl and incubating at 37°C for 2 h. Afterwards, another 4 μl of DNase I were added and incubated at 37°C for another 2 h. The RNA was then recovered and purified by phenol:chloroform followed by LiCl precipitation. The elimination of the DNA was confirmed by setting up a 2-step non-selective PCR [200 nM P1 primer, 200 nM P2 primer, 50 μM dNTPs in 25 μl total volume] using the RNA as template [40 pg/μl] and Taq Polymerase [0.025 U/μl]. Absence of a product indicated successful DNA elimination.

### HEK protein extract

A detailed protocol to obtain protein extract from HEK293T cells has been previously published (32). Briefly, HEK293T cells were plated in two 15 cm plates (5 × 10^6^ cells each) and transfected 24 h later with 5 μg of a Flag-hADAR2-pcDNA3.1(+) plasmid per plate using the Effectene Transfection Reagent Kit (Qiagen) or the TransIT-LT1 Transfection Reagent (Mirus). Four days post-transfection, the cells were washed with PBS, resuspended in ice-cold 1× TBS (50 mM Tris pH 7.5 and 150 mM NaCl) pooled, and centrifuged at 1500 × *g* for 5 min at 4°C. The cell pellet was washed with 1× TBS and treated with the NE-PER Extraction Reagents (ThermoFisher) supplemented with 1X HALT protease inhibitor (ThermoFisher) and following the manufacturer's instructions. The nuclear extract obtained was supplemented with glycerol and DTT (20% and 1 mM final concentration respectively), aliquoted, flash frozen in liquid N_2_ and stored at –80°C for further use. The presence of the hADAR2 in the extracts was confirmed by running a western blot using an anti-Flag antibody.

### hADAR2 purification

A detailed protocol to purify ADAR2 using anti-FLAG M2 magnetic beads (Sigma) from HEK293T cells has been previously published (32). Briefly, HEK293T cells were plated in two 15 cm plates (5 × 10^6^ cells each) and transfected 24 h later with 5 μg of a Flag-hADAR2-pcDNA3.1(+) plasmid per plate using the Effectene Transfection Reagent Kit (Qiagen) or the TransIT-LT1 Transfection Reagent (Mirus). Four days post-transfection, the cells were washed with PBS, resuspended in ice-cold 1× TBS (50 mM Tris pH 7.5 and 150 mM NaCl) pooled, and centrifuged at 1500 × *g* for 5 min at 4°C. The cell pellet was washed with 1× TBS and treated with 5 ml of lysis buffer (500 mM NaCl, 50 mM HEPES pH 7.4, 10% (w/v) glycerol, 1% Triton X-100) supplemented with 1× HALT protease inhibitor (ThermoFisher) and incubated on ice for 10 min. During this time, the lysate was passed through a 23^1/2^ G syringe needle several times to reduce viscosity before centrifuging it at 10 000 × *g* for 10 min at 4°C. The cleared supernatant was added to the anti-FLAG M2 magnetic beads, previously prepared according to the manufacturer's instructions, and incubated overnight at 4°C on a tube rotator. Afterwards, beads were washed thrice with 150 μl of wash buffer (1× TBS, 15% (w/v) glycerol supplemented with 1X HALT protease inhibitor), and the protein was eluted from the beads using a 3× Flag peptide in 125 μl of elution buffer (1× TBS, 15% (w/v) glycerol and 1X HALT protease inhibitor) and following the manufacturer's instructions. The purified protein was flash-frozen in liquid N_2_ and stored at –80°C for further use. The purity of the protein was assessed by running SDS-PAGE and staining the gel with GelCode Blue (ThermoFisher) (Supplementary Figure S8). In the same gel, a serial dilution of BSA standard was run to estimate the protein concentration using ImageJ (Supplementary Figure S8). The concentration of the purified hADAR2 was 228 ng/μl.

### *In vitro* editing assay

*In vitro* editing reactions were set up in Q200 buffer [50 mM Tris pH 7.0, 200 mM Potassium Glutamate, 20% (w/v) glycerol] supplemented with PMSF [5 mM], DTT [5 mM], tRNA [0.5 μg/μl], RNase inhibitor [1 U/μl] (NEB) and Protease Inhibitor (cOmplete™ EDTA-free, Roche). To promote inter-molecular over intra-molecular binding, the RNA substrate was diluted in water, incubated at 95°C for one minute and put back immediately on ice prior to adding it to a 2.25 nM final concentration in a 50 μl total reaction volume. Each substate was incubated with a serial dilution of hADAR2-HEK nuclear extract ranging from 0.3125 to 40 ng/μl at 32.5°C for 2 h. Afterwards, RNA was recovered by phenol:chloroform extraction followed by ammonium acetate precipitation.

From the RNA, cDNA was then synthesized using the AccuScript High Fidelity RT-PCR kit (Agilent) and used as template for Non-Selective PCR (described above). Confirmation of editing activity was done by Sanger sequencing.

### ARMS PCR

Once editing was confirmed by Sanger sequencing, edited molecules were recovered by ARMS PCR. cDNA obtained above was used as template in a 2-step PCR reaction [4 μl template/50μl reaction, 1 μM ARMS primer, 200 nM P2 primer, 50 μM dNTPs] using Vent(exo-) polymerase [0.02 U/μl]. For both substrates, the ARMS primer had a mismatch at position -1G (Supplementary Table S1). For the 21 nt substrate the PCR ran for 18 cycles with a 2 mM Mg^+2^ final concentration, while for the 31 nt substrate it was run for 22 cycles with a 2.5 mM Mg^+2^ final concentration. The ARMS PCR product was used directly as a template for the substrate regeneration process.

### Substrate regeneration

The regeneration process was done in three sequential PCRs. A PCR was set to amplify selected guides from the ARMS PCR product, followed by annealing and amplification PCRs described for the DNA construct assembly above. The guide amplification PCR [1 μl of 1:10 template dilution/40 μl reaction, 375 nM Regeneration primer, 375 nM P2 primer, 50 μM dNTPs] was set using Vent (exo-) polymerase [0.02U/μl]. For each substrate, a different regeneration primer was used (Supplementary Table S1). An annealing temperature of 60°C was used, and the PCR was run for 30 cycles. Afterwards, the guide amplicons were gel purified and used as template for the subsequent PCR reactions.

**Figure 2.**
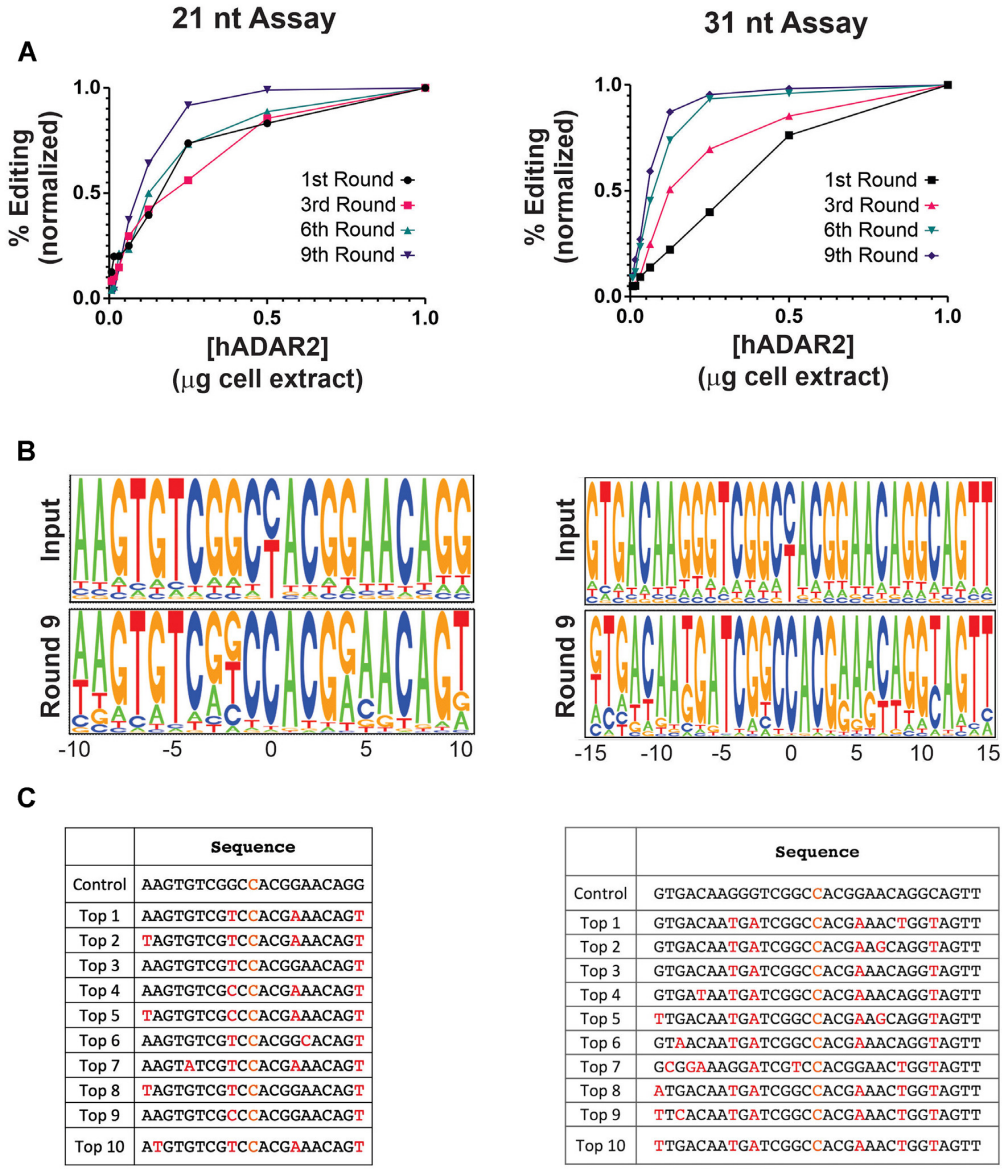
Evaluation of the selection assay. (**A**) Percent editing of the hairpin pool at different hADAR2 concentrations for rounds 1, 3, 6 and 9 (products after step 4 in Figure 1). For comparison purposes, percent editing was normalized to the highest value, and hADAR2 concentrations were normalized to the amount of cell extract that produced the highest editing per substrate per round. *n* = 1. (**B**) Logo plots showing the base preference at each position of the Guide region in the pool of hairpins (products after step 6 in Figure 1). Input refers to the starting material prior to the first round of editing. Position 0 marks the position complementary to the target adenosine. (**C**) Sequences of the Guide region for the top 10 most abundant hairpins after nine rounds of selection. Bases in red mark mismatches except for the C mismatch opposite the target A, which is marked in orange. Left panels correspond to the assay run with the 21 nt substrate and the right panels correspond to the assay run with the 31 nt substrate.

As an alternative, we engineered a restriction site at the end of the target region to remove it by enzymatic digest instead of PCR. A disadvantage of this approach was that due to the introduction of the restriction site, there was a difference of one base between the 21 nt and the 31 nt sequences at position –7 (Figure 2B). This difference limited the direct comparison of results between the two substrates. Though we reasoned that removing the edited target region using restriction enzymes would reduce the error introduced in the system by multiple PCRs with non-proofreading polymerases in the different rounds of selection, the ARMS PCR did not yield enough material for running an efficient digest and DNA purification. In contrast, we did not observe an increase in the number of reads that had more than 6 mismatches outside the Guide region (Supplementary Tables S2 and S3). Therefore, removing the edited target while amplifying the guide region through PCR was the better alternative.

### Bioinformatic analysis

Illumina adaptors were added to the regenerated DNA constructs by a 2-step PCR [0.4 μl of 1:100 template dilution/40 μl reaction, 375 nM forward primer, 375 nM reverse primer, 50 μM dNTPs] using Vent(exo-) polymerase [0.02 U/μl] and MiSeq primers (Supplementary Table S1). The amplicon was sequenced using the Amplicon-EZ (paired-end MiSeq) service from Azenta Life Sciences. The obtained reads were aligned to the reference DNA sequence using Bowtie2 with local configuration and default parameters (33). Aligned reads that were not paired or that mapped more than once were filtered out using the SAMtools package (34). The remaining reads were further filtered to eliminate reads with InDels and reads that had more than six mismatches (maximum number that can occur by chance assuming a sequencing error rate of 0.1%) in places other than the guide region in comparison to the reference sequence. The remaining reads were fed into Python scripts developed to determine the guide sequence diversity, the base preference per position, the mismatch distribution, and the enriched guide sequences.

### *In vitro* hairpin testing

RNA substrates containing the top 10 enriched guide sequences were synthesized as described above. Editing reactions were set up as described above, using two concentrations (5 nM and 40 nM in 50 μl total volume) of purified ADAR2 protein. Editing percentages were determined by RT-PCR and Sanger sequencing as described above.

### *In vitro* hairpin editing kinetics

A single editing reaction per RNA substrate containing the selected guide was set up as described above. To ensure single turnover conditions and good resolution at early time points, the concentration of purified ADAR2 used was 80 nM and 30 nM for the 21 nt and 31 nt substrates respectively. Prior to adding the enzyme, the reaction mixes containing the annealed RNA were equilibrated at 32.5°C for 10 min. After adding the enzyme, aliquots were taken at 0, 1, 2, 5, 10, 20, 40, 80 and 120 minutes and put directly into phenol:chloroform to stop the reaction. Editing percentages were determined by RT-PCR and Sanger sequencing. The obtained values were fitted to a one-phase association curve [*E* = *E*_max_(1 – e^−*kt*^)] to determine the rate constant, *k*.

### *In vitro* top guides testing (*in trans)*

In Q200 buffer supplemented with PMSF and protease inhibitor, *in vitro* transcribed mCherry-eGFPW58X target RNA was mixed with chemically synthesized guide RNAs at final concentrations of 2.25 and 22.5 nM, respectively. To anneal the guide to the target, the RNA mixture was incubated at 75°C for 1 min and cooled to 25°C at a rate of 0.1°C/s. After a 1 min incubation at 25°C, the reaction was supplemented with DTT, RNase Inhibitor and tRNA. Purified ADAR2 protein was then added (5 nM final concentration in 50 μl total volume) and the reactions were incubated at 32.5°C for 10 min. Editing percentages were determined by RT-PCR and Sanger sequencing as described above. For determining the kinetic rates, single turnover conditions and good resolution at early time points were achieved by setting the concentration of purified ADAR2 to 80 nM and 10 nM for the 21 nt and the 31 nt substrates respectively. Prior to adding the enzyme, the reaction mixes were equilibrated at 32.5°C for 10 min. After adding the enzyme, aliquots were taken at 0, 1, 2, 5, 10, 20, 40, 80 and 120 min and put directly into phenol:chloroform to stop the reaction. Editing percentages were determined by RT-PCR and Sanger Sequencing or MiSeq. The obtained values were fitted to a one-phase association curve [*E* = *E*_max_(1 – e^−*kt*^)] to determine the rate constant, *k*.

### Cell culture

ADAR1 knockout HEK293T cells were obtained from GenScript by deletion of 10–16 bp using CRISPR. The obtained cells were grown in DMEM (Corning, Catalog number: 10-017-CV) supplemented with 10% FBS (Gibco, Catalog number: 26140-079) in an incubator at 37°C and 5% CO_2_.

### *In cellula* editing assays with oligonucleotides

ADAR1 knockout HEK293T cells (5 × 10^6^ cells/10 cm^2^ plate) were co-transfected with huMycADAR2, huMycADAR1-p110 or huMycADAR1-p150 (2μg), and mCherry_2PA_eGFP(W58X)_pcDNA3.1(–) (10μg) plasmids to express human ADAR2, ADAR1-p110 or ADAR1-p150, and eGFP containing the early termination mutation W58X. After 24 h, cells were detached with 0.05% trypsin-EDTA (Gibco, Catalog number: 25300-054) and 5000 cells/well were reverse transfected with oligonucleotides in 384-well plates. Both plasmid and oligonucleotide transfections were performed with Lipofectamine 3000 (ThermoFisher, Catalog number: L3000-015) diluted in OptiMEM (Gibco, Catalog number: 11058–021) following the manufacturer's instructions. After 48 h, eGFP fluorescence was imaged using an EVOS M7000 microscope (Invitrogen) and quantified with a BioTek Cytation5 imaging reader with excitation/emission wavelengths at 488 nm/510 nm, respectively, before cells were lysed and mRNA was isolated using Dynabeads™ Oligo(dT)_25_ (ThermoFisher, Catalog number: 61005). Isolated mRNA was cleaned with ezDNase to avoid genomic and plasmid DNA contamination and then reverse transcribed to cDNA with SuperScript IV Vilo (ThermoFisher, Catalog number: 1176500) before proceeding to next generation sequencing (NGS).

### Analysis of RNA editing

NGS libraries were prepared and sequenced at Quintara Bioscience (Cambridge, MA) using a 2-step PCR approach. First, amplicons were generated using primers containing a random hexamer UMI and Illumina sequencing adapters. After cleanup, unique dual index combinations were added to each sample in a second PCR reaction. Samples were then pooled and sequenced on 2 × 150 PE runs on the MiSeq platform, and demultiplexed using standard Illumina software. UMIs were detected for each paired-end read in the fastq files using UMI_tools extract (35). Low quality bases and adapter sequences were then trimmed using Trim Galore! with default settings (36). Overlapping paired-end reads were collapsed into a single read using leeHom (37). Successfully merged reads were then aligned to the reference sequence using bwa mem (38). Unmapped reads, secondary alignments and reads with mapping quality <30 were filtered using SAMtools (34), and deduplicated using umi_tools dedup (35).

Editing was calculated from bam files using a custom suite of python tools. Briefly, for each sample, deduplicated reads were used to determine base counts at the target site, and editing was calculated as the ratio of G/(G + A). An empirical p-value for editing in each sample was calculated using kernel density estimation over the frequency distribution of errors across the amplicon.

### Potency determination

For potency determination, 5000 ADAR1 knockout HEK293T cells expressing human ADAR2 and eGFP-W58X were reverse transfected with oligonucleotides as described above with varying concentrations (from 0.07 to 300 nM). RNA editing was analyzed with NGS as described above. The resulting editing percentages and EC_50_ values were calculated using GraphPad Prism 9.

## RESULTS

### Development of an assay to select gRNAs to drive RNA editing

Any assay designed to select optimized ASOs to drive SDRE is confronted by two main challenges. The first is that for SDRE, ASOs generate an editable structure *in trans*, unlike the case for naturally edited structures, which form *in cis*. The efficacy of an ASO is generally determined by sequencing the edited target; thus, when one is screening large pools of ASOs, successful outcomes cannot be linked to the ASOs that produced them. The second challenge is the vast mutational space that must be covered, even for small ASOs. There are a few generalized rules for gRNA design that reduce the space. For example, a nominally double-stranded structure is required, and a C opposite the target A improves editing efficiency (39), but still the space remains very large. To meet these challenges, we designed an assay where the ASO gRNAs and the editing target are *in cis*. In addition, we limited the mutational space to ASOs that are mostly base-paired, containing ≤25% mismatched positions.

We focused our selection efforts on two ASO lengths, 21 nt and 31 nt, both similar in length to FDA approved oligonucleotides (21,22). To create the editable RNA substrate with the target and the guide *in cis*, we started with two DNA oligos, one encoding the ‘target’ and the other encoding the ‘guides’ (Figure 1, Supplementary Table S1). As a target, we chose 20 or 30 nt symmetrically surrounding the editable A in an engineered premature termination codon in the eGFP transcript (W58X; UGG→UAG). This target was chosen as it would allow a facile assessment of both mRNA and protein level correction by the selected gRNAs in cells. To avoid the complication of off-target edits in the target region, A's at silent positions were changed to G's. Upstream of the target region the construct contains a T7 promoter for *in vitro* transcription and a universal forward primer (P1, Supplementary Table S1) used for RT-PCR and deep sequencing. Downstream of the target region lies the sequence for a 25nt connector loop that is predicted by *in silico* folding analyses (RNAfold) to be unstructured.

Random guide sequences are introduced via the ‘Guide’ oligo. The ‘Guide’ oligo contains 21 or 31 nt sequences that are mostly complementary to the corresponding eGFP targets, with the caveat that during synthesis, 91% of each position is the complementary base, and 3% each of the other three bases. The 91:3:3:3 randomization scheme ensured that the majority of molecules would form dsRNA. However, one consequence of choosing this level of randomization is that the screening is skewed towards sequences with a low number of mismatches. For example, for the 21 nt target, 99% of the molecules contain 0-5 mismatches and for the 31 nt target, 99% would have 0–7 mismatches. As an internal control for selection, a 50:50 C:T mix was placed at the position opposite the target A as it is well known that a C mismatch improves editing. Thus, we would expect C to be selected at this position over the course of the assay. The loop sequence was used to connect the Guide and Target oligos together. Downstream of the randomized portion there is a partial sequence for a reverse primer (P2, Supplementary Table S1) used for RT-PCR and Deep Sequencing.

A single DNA construct comprising both the target and the guide regions is then generated by PCR (Figure 1, Step 1; the complete P2 primer is also formed during this step). RNA hairpin substrates are then synthesized from this template by *in vitro* transcription (Step 2). The hairpins are then edited *in vitro* using extract from HEK293T cells over-expressing human ADAR2 (Step 3). We elected to use ADAR2 as it catalyzes most recoding events in mammals (40,41). This reaction produces edited and unedited products, and the probability of an edited outcome is proportional to the efficacy of the accompanying gRNA sequence. At the end of the editing reaction, the RNA is recovered and reverse transcribed (Step 4). The cDNA obtained is used as a substrate for two different PCRs. First, a non-selective PCR is performed using the P1 and P2 primers, where Sanger sequencing of the product monitors the editing efficiency at each round of the assay. Second, a selective PCR using ARMS primers is performed (Supplementary Table S1) to isolate the population of guides that promote editing of the target A (Step 5). The conditions for exclusively amplifying edited sequences using ARMS PCR were determined on control substrates (Supplemental Information, Supplementary Figure S1). The ARMS PCR products are used as a template for a PCR that creates a new version of the Guide oligo (Step 6) which only contains the guides that promote editing. This step both reduces the diversity of the guides being tested each round and, enriches the guides that promote efficient editing. The new population is then annealed to the original Target oligo and the product is used as the starting point for the next round of selection. Here, the enriched Guide pool is subject to single molecule sequencing (MiSeq) tracking the progress of selection.

### Selection assay evaluation

The selection that drives the assay comes from ADAR2’s ability or inability to edit the substrate. In theory, the selective pressure could be calibrated in several ways. In setting up the assay, we hypothesized that running it under stringent conditions (e.g. a low enzyme concentration or a short incubation time) would help to select for the most efficient guide sequences. We chose to use a low enzyme concentration as the only selective pressure. Editing reactions were carried out for 2 hours using serial dilutions of ADAR2 protein extracts (ranging from a 1 to a 1/128 dilution) and editing efficiencies were calculated from Sanger sequencing electropherograms. For both the 21 and 31 nt substrates, editing efficiencies plateaued at higher protein concentrations (Figure 2A). For subsequent substrate regeneration, we chose the cDNA template from the ADAR2 dilution in which ∼5% editing was observed (1/32 dilution). In addition, these dilutions allowed us to monitor editing efficiency at each round of the assay. If the assay was selecting more efficient guides, we would expect the editing efficiency to increase at each round. For both the 21 and 31 nt substrates, editing generally improved at each round and this was more evident with intermediate protein levels. For the 31 nt substrate, the improvement was more apparent than for the 21 nt substrate (Figure 2A, Supplementary Figure S2A). For both substrates, rounds 7, 8, and 9 produced similar levels of editing and a similar concentration dependence (Supplementary Figure S2A). Accordingly, the assay was stopped after 9 rounds.

The progress of the assay was monitored in greater detail using low coverage MiSeq (∼5 × 10^4^ reads/sample). These data allowed us to not only examine positional base preference within the guide region, but also the individual guide sequences that promoted the most efficient editing. Position 0 within the guide region (opposite to the target A), for both the 21 nt and 31 nt, which started the assay as an equimolar mix of C and T, served as an internal control for selection. It is well established that ADARs prefer a C at this position, and a C was indeed selected after the third round for both substrates (Figure 2B, Supplementary Figure S3). At many positions, the selected pool of guides trended towards the complementary base. This was particularly apparent at positions –5, –4, –1, +1, +2 and +3 for both guide lengths, indicating that formation of dsRNA close to the target editing site might be necessary for efficient editing. The positions where the complementary base was not preferred, on the other hand, rarely showed a strong preference for a single base, although T mismatches were frequently encountered. For the 21 nt guide, position +4 showed an almost equal preference for A and G, while position –2 showed similar preference for G, T or C. In this substrate, a strong preference for a non-complementary base was observed only at position +10. In the 31 nt guide, 3 positions (–8, –6 and +11) showed a strong preference for a non-complementary base. The overall results for both the 21 nt and 31 nt substrates showed that many of the bases surrounding the editing site must be complementary; however, at positions further from the editing site, the base preference depended on the length of the gRNA.

From the MiSeq data we were also able to determine how the overall guide population changed round by round. For the 21 nt substrate, there was a clear reduction in guide diversity, as the number of reads representing unique sequences was reduced from 53.9% in the original unedited construct (control) to 21.9% after nine rounds of selection (Supplementary Table S2). For the 31 nt substrate, a significant reduction in overall guide diversity was not observed (72% of the reads were unique sequences in both the original substrate and after round 9, Supplementary Table S3). This result could be due to the vast starting mutational space and the relative low depth of MiSeq coverage (for the 31 nt substrate there were 1.15 × 10^18^ potential sequences, and only 1.10 × 10^12^ for the 21 nt substrate, a difference of six orders of magnitude). MiSeq data, at ∼5 × 10^4^ reads per sample, is a random sampling of a subset of the guides present and thus represents only a snapshot of the true diversity. Though using a different platform with deeper coverage (e.g. HiSeq) would have given us a better idea of the total diversity of the population, for the purpose of detecting individual sequences that were getting enriched during the selection rounds, the MiSeq depth was adequate.

From the MiSeq data, we ranked the frequency at which specific guide sequences were encountered in the overall pool. Since we determined the conditions in which ARMS PCR would only amplify edited substrates (Supplemental Information, Supplementary Figure S1), we hypothesized that only guide sequences that promoted efficient editing would get enriched over the rounds, regardless of the initial representation of the sequence in the initial pool. By tracking individual sequences within this data, we observed that the representation of the most frequently encountered sequences increased gradually by round (Supplementary Figure S2B). The ten most enriched sequences at the end of the assay (round 9) were not encountered during the early rounds. For the 21 nt substrate, some of the enriched sequences could be seen after round 3, and most after round 6. Interestingly, for both substrates, the number of reads for a perfectly complementary guide sequence with an A/C mismatch opposite the target A decreased by round. In particular for 31 nt, the control guide went from being the most represented sequence at the outset to being undetectable after round 9. These results confirm that perfectly complementary guides were not ideal and that guides that promoted more efficient editing were enriched, while the less efficient guides were eliminated.

For the 21 nt substrate, the top 10 most frequently encountered sequences had between 2 to 4 mismatches (in addition to the A/C mismatch, Figure 2C). All of them contained a mismatched T at position +10. Similarly, for the 31 nt substrate, the top 10 most represented sequences had 4–7 mismatches (Figure 2C). All of them showed the same mismatches at positions –6 and + 11, suggesting that A and T at those positions are desirable for editing. In fact, most of the top 10 sequences are very similar, with variations at two or three positions. The seventh most frequently encountered sequence (Top 7) was an exception, showing relatively little similarity to the others. This suggests that within the guide sequences, some positions have strong preferences for specific bases while others are more flexible.

### Testing editing efficiencies of the most frequently encountered hairpins *in vitro*

**Figure 3.**
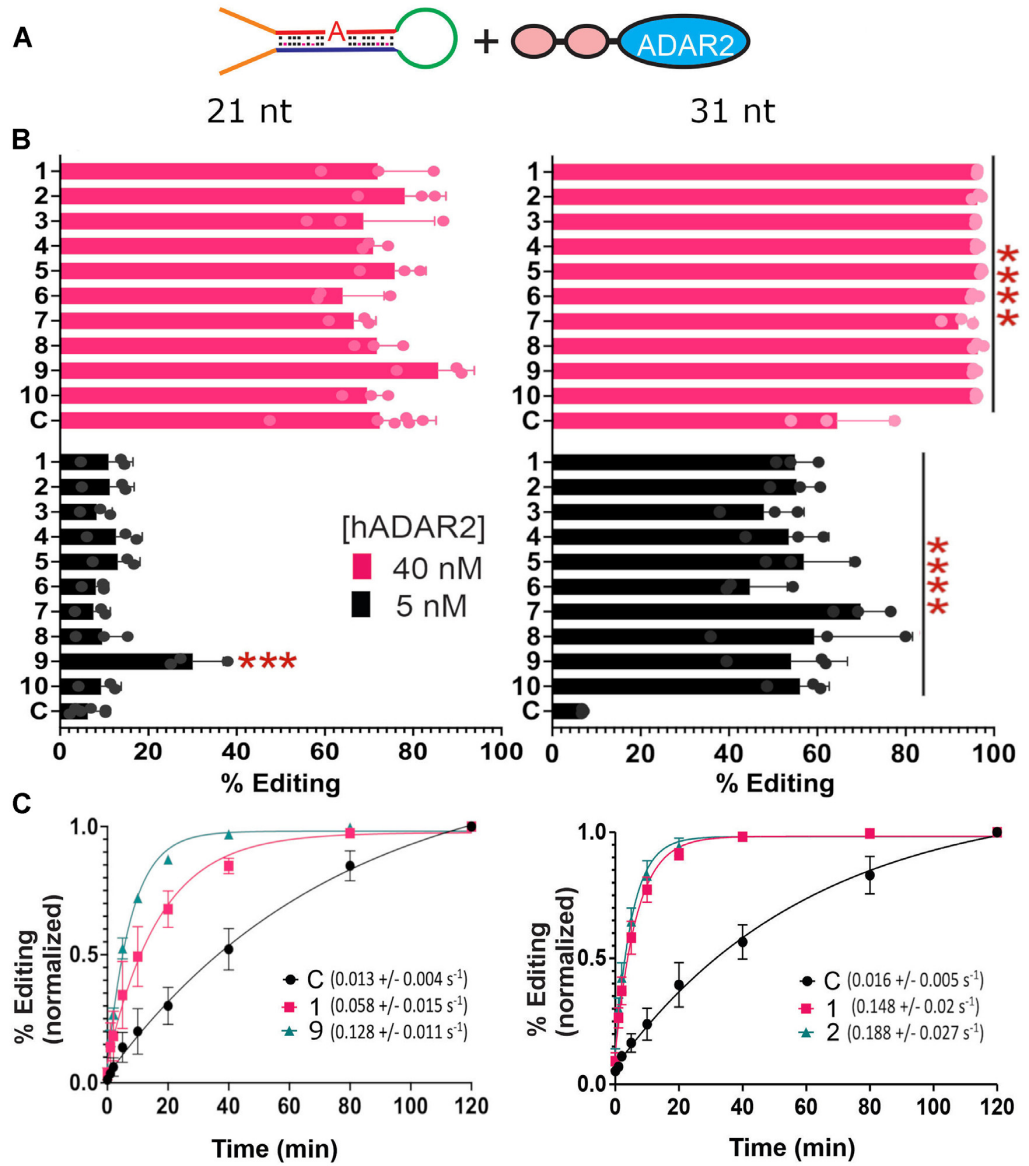
Testing top hairpins *in vitro*. (**A**) A cartoon showing *in vitro* editing reactions using individual hairpins containing one of the 10 most abundant guide sequences and with purified hADAR2. (**B**) Percent editing of the top 10 most abundant hairpins using 40nM (pink) or 5nM (black) concentrations of hADAR2. Asterisks denote a statistically significant difference in comparison to the control hairpin (two-way ANOVA, **** *P* < 0.0001, *** *P* < 0.001, for 21 nt control *n* = 6, for all other samples *n* = 3). Error bars represent S.D. (**C**) Reaction kinetics for two of the most abundant hairpins and the control hairpin under single turnover conditions. Values in parenthesis are the respective rate constants in s^−1^. For comparison purposes, percent editing was normalized to the highest value for each hairpin. Data were fit to a one-phase association curve (see methods). Error bars represent S.D., *n* = 3.

Based on the selection assay, the most frequently encountered guide sequences identified after nine rounds of selection are predicted to drive superior editing. We performed all further downstream analysis on the top ten sequences from round nine of the 21 nt and 31 nt selection assays. First, we synthesized RNA hairpins identical to those used in the selection assay (Figure 1 Step 2) where the guide region was fixed as one of the top 10 most frequently encountered sequences. In this configuration the guide and target regions are still *in cis*, as they were during selection. These constructs were edited *in vitro* with fixed concentrations of human ADAR2 purified from HEK293T cells (Figure 3A). A perfectly complementary guide with an A/C mismatch opposite the target A was included as a control. For the 21 nt substrate, only one of the guides exhibited better editing in comparison to the control (Figure 3B). At 5 nM, editing with the control appears to be less complete in comparison to the selected guides, but there was only significant difference in comparison to the Top 9 guide (Figure 3B). To compare the selected 21 nt guides in greater detail, we also measured reaction kinetics under single turnover conditions (Figure 3C). For these experiments, we selected the Top 1 and Top 9 guides as comparators for the control. Both selected guides drive faster reaction kinetics than the control. In fact, the rate constants for the Top 1 (0.058 ± 0.015 s^−1^) and Top 9 (0.128 ± 0.011 s^−1^) are about 4 and 10-fold faster than the control (0.013 ± 0.004 s^−1^) and these differences were statistically significant. From this we conclude that the selected guides had superior kinetics, but this difference became less significant when enough reaction time passed to reach the steady state conditions of the initial measurements.

In contrast to the 21 nt selected guides, all top 10 guides from the 31 nt selection showed significantly higher editing in comparison to the control at both high and low enzyme concentrations (Figure 3B). The difference was more pronounced at 5 nM than at 40 nM, most likely because the reactions for the selected guides were close to saturation with the higher enzyme concentration. We next examined the reaction kinetics for the Top 1, Top 2, and control hairpins for the 31 nt selection (Figure 3C). The kinetics of both top guides were significantly faster than the control. The rate constants for the Top 1 guide (0.148 ± 0.02 s^−1^) and Top 2 guide (0.188 ± 0.027 s^−1^) were 9.2 and 11.7-fold faster than the value for the control (0.016 ± 0.005 s^−1^), respectively. Interestingly, though the control guide for both 21 nt and 31 nt substrates had similar rate constants, the top guides from the 31 nt selection drove much faster reactions than those for the 21 nt selection. These data suggest that the selection strategy enriched for hairpin sequences with faster kinetics in comparison to the perfectly matched controls, and this was most pronounced for the 31 nt substrates.

### Testing editing efficiencies of the most frequently encountered gRNA sequences *in vitro*

**Figure 4.**
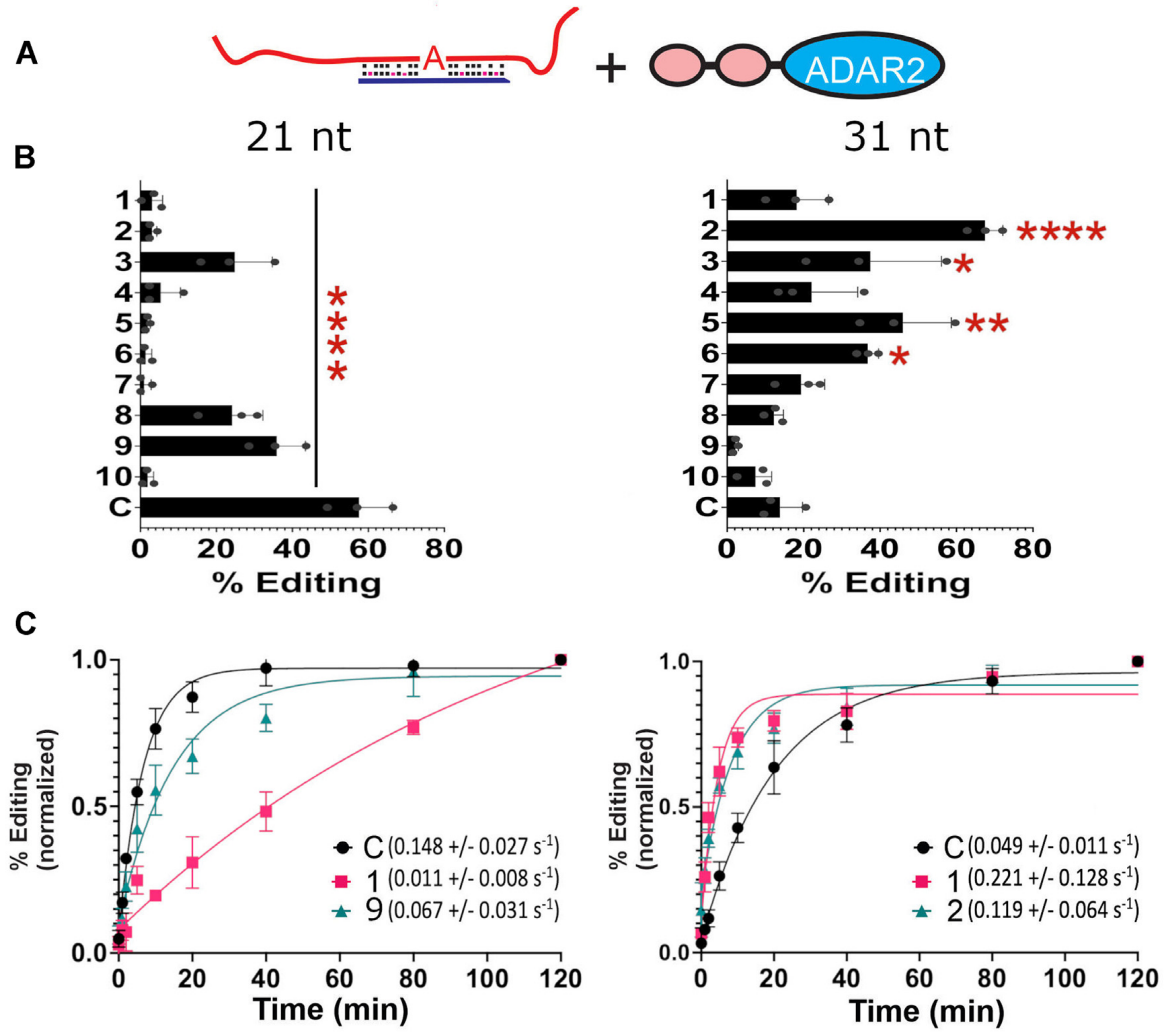
Testing top guide RNAs *in vitro*. (**A)** A cartoon showing *in vitro* editing reactions using individual gRNAs (*in trans*) of the 10 most abundant guide sequences with purified hADAR2 and full length mCherry_P2A_eGFP W58X (UAG) mRNA. (**B**) Percent editing of the top 10 most abundant gRNAs at a 5nM concentration of hADAR2. Asterisks denote a statistically significant difference in comparison to the control gRNA (one way ANOVA, **** *P* < 0.0001, ** *P* < 0.01, *P < 0.05, *n* = 3). Error bars represent S.D. (**C**) Reaction kinetics for two of the most abundant gRNAs and the control gRNA. Values in parenthesis are the respective rate constants in s^−1^. For comparison purposes percent editing was normalized to the highest value for each gRNA. Data were fit to a one-phase association curve (see methods). Error bars represent S.D., *n* = 3.

Because SDRE relies on guide oligonucleotides that form editable structures *in trans*, we tested whether the guide regions from our selected hairpins would drive superior editing when delivered *in trans* (Figure 4A). Target RNAs were transcribed *in vitro*, while the gRNAs were chemically synthesized, and editing reactions were set up *in vitro* with a low concentration of recombinant hADAR2 in order to maximize differences. In all cases, the editing efficiency varied widely between the guides. For the 21 nt guides, all performed significantly worse than the control (Figure 4B). For the 31 nt guides, guides 2, 3, 5 and 6 performed significantly better than the control. Guides 1, 4 and 7 to 10 were not significantly different than the control. Reaction kinetics were also measured for two guides and the control from each group (Figure 4C). For the 21 nt guides, we selected a poorly performing guide (Top 1) and a better performing guide (Top 9). The reaction rate constant of the control guide (0.148 ± 0.027 s^−1^) was 13.4-fold higher than the rate for the Top 1 guide (0.011 ± 0.008 s^−1^) and 2.2-fold higher than Top 9 guide (0.067 ± 0.031 s^−1^).

For the 31 nt guides, we also selected a poorly performing guide (Top 1) and a well performing guide (Top 2, Figure 4C). Interestingly, though the Top 1 guide did not show significantly different steady-state editing in comparison to the control (Figure 4B), its rate constant (0.221 ± 0.128 s^−1^) was 4.5-fold higher than the control (0.049 ± 0.011 s^−1^). Furthermore, the Top 2 guide had a rate constant (0.119 ± 0.064 s^−1^) 2.4-fold higher than the control but less than that for the Top 1 guide. These data suggest that the editing efficiency of a guide *in vitro* is not solely determined by the reaction rate constant and other factors (e.g. annealing) might affect the overall efficiency. Furthermore, the removal of the loop appeared to reduce the editing efficiency and this effect was most pronounced for the 21 nt substrates.

### Evaluation of guide activity in HEK293T cells

After *in vitro* testing of the 21 nt and 31 nt guides, we next tested their efficacies *in cellula*. For these experiments we elected to use ADAR1 ^−/−^ HEK293T cells because HEK293T cells show inherently low endogenous ADAR2 expression and the further elimination of ADAR1 expression makes them useful for examining editing driven by the overexpression of any active form of human ADAR (hADAR1-p110, hADAR1-p150, hADAR2). A plasmid encoding mCherry_P2A_eGFP W58X (UAG) was co-transfected into these cells with plasmids encoding either hADAR1-p110, hADAR1-p150, or hADAR2. Confirmation of target plasmid expression was achieved by monitoring the red fluorescence produced by mCherry. To correct the eGFP W58X premature termination codon, gRNA ASOs were directly transfected into the cells at a single dose of 100nM. Successful editing of the W58X stop codon could be monitored at the RNA level by NGS, or by monitoring the production of green fluorescence.

**Figure 5.**
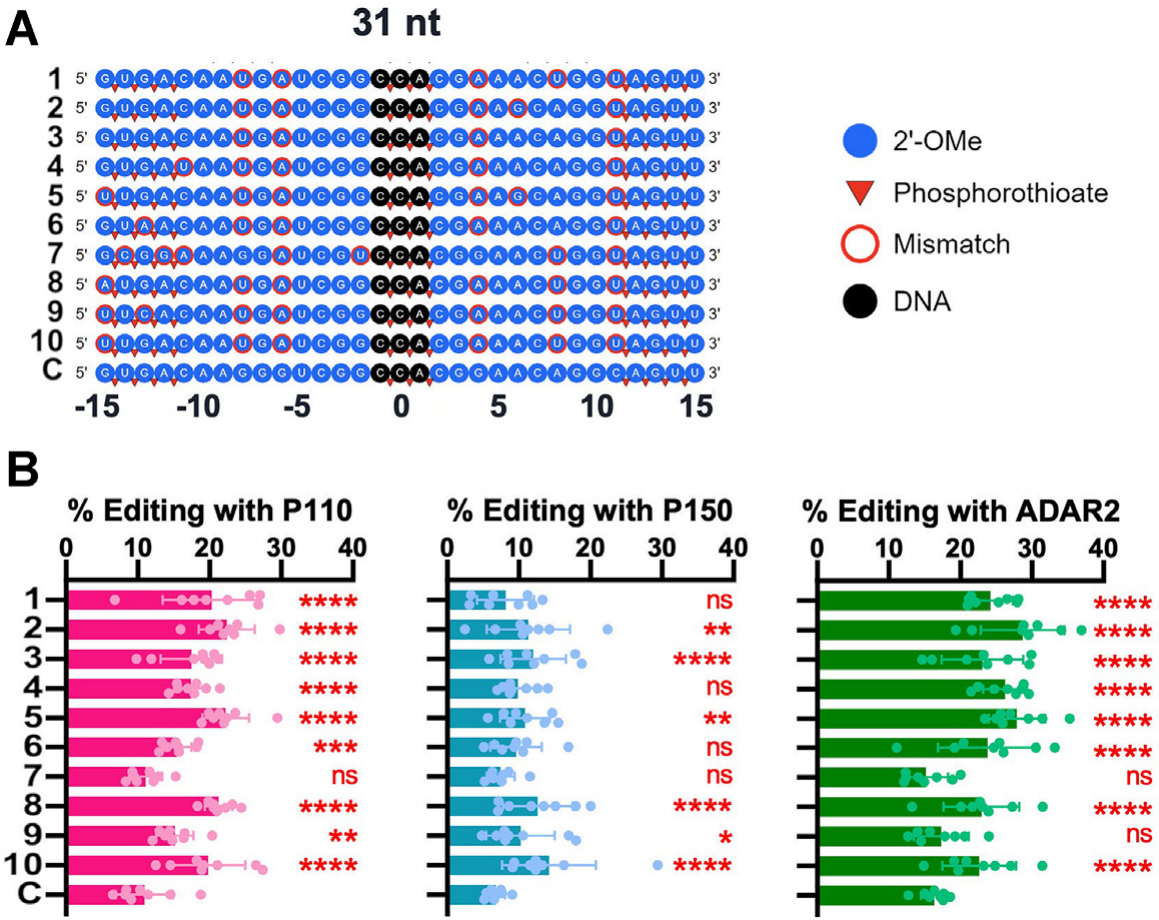
Testing top guide RNAs *in cellula*. (**A**) A map of the chemical modifications within the stabilized 31 nt ASOs of the 10 most abundant gRNAs as well as the control gRNA sequences. ASOs (100 nM) were transfected into HEK293T cells transiently expressing mCherry_P2A_eGFP W58X (UAG) and hADAR1-p110, hADAR1-p150 or hADAR2. (**B**) Percent editing of the top 10 31 nt gRNAs from the selection assay in comparison to the control. Asterisks denote statistically significant differences in comparison to the control gRNA (Two-way ANOVA, **** *P* < 0.0001, *** *P* < 0.001, ** *P* < 0.01, *P < 0.05, n.s. not significant). Error bars represent S.D., and for all data points, *n* = 8.

Chemical stabilization is generally preferred for RNA ASOs directly transfected into the cellular environment because of endogenous nucleases and phosphatases. The initial stabilization strategy introduced four phosphorothioate backbone modifications to both 5’ and 3’ ends of gRNAs (Supplementary Figure S4A). These guides showed very low activity in HEK293T cells, suggesting the stabilization was insufficient (Supplementary Figure S4B). For both the 21 nt gRNAs with either hADAR1-p110, hADAR1-p150 or hADAR2, editing was generally less than 1%, and for the 31 nt gRNAs it was less than 2%. These low editing percentages made it difficult to judge the efficacy of selected gRNAs *vs*. controls. To further improve the gRNAs’ stability, we fully modified the 21 and 31 nt guides. In addition to the phosphorothioate stabilization of the 5’ and 3’ termini, we converted the 2’-OH to 2’-OMethyl (2’-OMe) for all nucleotides except for the triplet at the center of the sequence, which was converted to DNA (Figure 5A and Supplementary Figure S4C). The central DNA triplet was further stabilized using three phosphorothioate linkages. These chemical modifications are commonly utilized in approved oligonucleotide drugs (42).

Similar to the *in vitro* results (Figure 4), the 21 nt guides behaved poorly compared to the perfectly complementary control (Supplementary Figure S4D). In each case, using hADAR1-p110, hADAR1-p150 or hADAR2, the control outperformed the selected gRNAs, where the control performed poorly as well (generally < 10%). These data support the hypothesis that the 21 nt guides may be too short to drive efficient editing activity and that the loop connecting the target and the gRNA may have contributed to selection in the selection assay. In contrast, the majority of 31 nt guides performed better than the control with all three ADAR isoforms (Figure 5B). The best activity overall was seen in conditions with hADAR2 overexpression, which could reflect the use of hADAR2 in the selection screen. The Top 2 guide showed the most improvement, with 1.8× activity compared to the parent control (28.7% versus 16.4%). These experiments were performed with a single concentration of ASO (100 nM).

**Figure 6.**
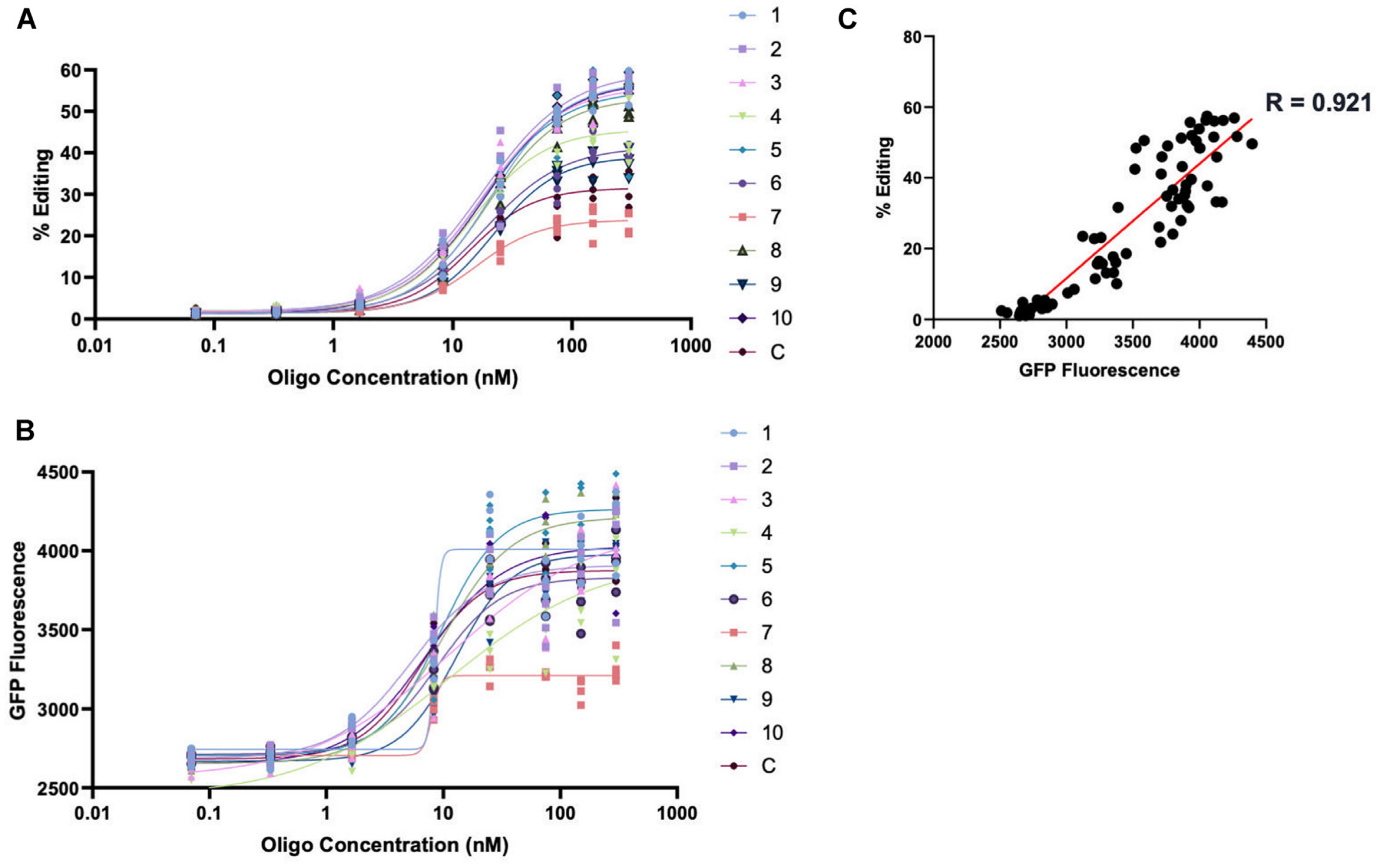
Dose response of the stabilized 31 nt ASOs of the 10 most abundant gRNAs *in cellula*. (**A**) Percent editing at different concentrations of the top 10 most abundant gRNAs in comparison to the control after HEK293T cells co-transfection with plasmids encoding mCherry_P2A_eGFP W58X (UAG) and hADAR2. A four-parameter non-linear plot was used to fit the data. For all data points, *n* = 4. (**B**) GFP fluorescence at different concentrations of transfected gRNAs shown in (A). A four-parameter non-linear plot was used to fit the data. For all data points, *n* = 4. (**C**) Linear regression showing the correlation between percent editing and GFP fluorescence.

To further characterize the potency of the selected 31 nt guides, a dose–response curve was generated using the Top 10 guides and hADAR2 (Figure 6A). Interestingly, while the EC_50s_ of the different gRNAs were similar across the Top 10 guides (13.9–21.8 nM), the maximum editing achieved varied substantially (Figure 6A, Supplementary Figure S5). Maximal editing with the control gRNA was 32%, a value that was below all the selected gRNAs except the Top 7 guide. The gRNAs Top 2, Top 3, Top 5 and Top 10 all drove editing at 56–57%. Editing was also quantified across all gRNAs and doses at the protein level by measuring eGFP fluorescence (Figure 6B, see Supplementary Figure S6 for an example of images across doses). In general, the relative gRNA activities followed a similar pattern as with the RNA-level testing. In addition, we noted a significant correlation between RNA editing and GFP intensity values (Figure 6C).

## DISCUSSION

As diverse systems for SDRE are developed, a central question is whether there are general rules for gRNA design. Although small gRNAs are more likely preferred as therapeutic agents, determining rules for their design is particularly challenging because the sequence which can be manipulated to create editable structures is limited. Pioneering studies on ADARs and the structures that they prefer have helped to identify features that promote editing. For example, a mismatched C opposite the target A, or a 5’ U or a 3’ G surrounding the target A are beneficial. Neighboring sequence motifs further out can also influence editing, as do the presence of > 6bp loops in extended structures (43). In contrast to naturally occurring ADAR substrates which form *in cis*, SDRE applications impose limitations on the creation of editable structures. Because the structure is formed when an ASO is delivered *in trans*, only the sequence of the ASO can be manipulated. Influential neighboring residues to the target A cannot be controlled, and if designs are limited to small oligos, large, naturally occurring structural elements (e.g. the R/G site structure of GluR2) cannot be incorporated. Thus, at the outset of this work, it was unclear whether small gRNAs that promote efficient editing could be designed at all.

Our results indicate that our selection assay was effective at selecting superior small hairpins that promote editing. Only in the case of the 31 nt substrate did the guide region of some of these hairpins translate into superior editing when delivered *in trans*. These data indicate that sequences outside of the complementary guide region played a role during the hairpin selection, with the connector loop being an obvious candidate. Interestingly, while the 21 nt and 31 nt substrates targeted essentially the same sequence, the selected harpins had different compositions, particularly with regards to the positions of mismatches (Figure 2B and C) with the exception of a common A mismatch at position 4. In general, T's and A's were the preferred residues when mismatches occurred. On the other hand, some positions did not support mismatches and clearly converged on complementarity, particularly at positions close to the target A (e.g. positions –1, 1, 2 and 3). The efficacy of having a C mismatch at position 0 was unambiguously supported by the selection.

Although there were similarities for the 21 nt and 31 nt gRNAs in base preference near the target region (*i.e*. positions –5 to +5), the two sets of guides performed quite differently. In most cases for the 21 nt selection, the guides edited poorly *in vitro* and *in cellula* (Figure 4 and Supplementary Figure S3). This contrasted sharply with their performance as hairpins (Figure 3). This may suggest that a length of 21 nt is insufficient to create an effective editable structure, even though small editable structures have been observed before. For example, the smallest editable substrate reported so far was 17 bp long, though editing with this substrate was ∼3 fold less efficient than when the structure was extended to 23 bp (44). Though 23 bp substrates can be edited (39,45–46), they are edited more efficiently at high enzyme concentrations (45) or by an ADAR2 construct that lacks the dsRBDs (39). Work from the Beal lab has shown that extending a 23 bp substrate by 5 bp on the 3’ end allows it to accommodate the second dsRBD of ADAR2 (47). This result suggests that any editable structure may need to be at least 28 bp long to be efficiently edited by endogenous ADAR2, further supporting the idea that a 21 nt gRNA may be too short. Further studies will be required to assess the minimal length of effective gRNAs.

In contrast to the 21 nt guides, 31 nt guides should be long enough to generate editable structures. The 31 nt hairpins selected by our assay all showed superior editing to the control; however, when the guide regions from the top 10 of these hairpins were tested *in trans in vitro* (Figure 4), only four promoted better editing than the control (the other six were statistically similar). A greater portion of the guides performed better *in cellula* with transfected ADARs. Under these conditions, 8 guides performed better than the control with hADAR2, 9 with hADAR1-p110 and 6 with hADAR1-p150 (Figure 5). Unlike the guides that were used *in vitro*, those used for the *in cellula* assays were modified with 2’-OMe, phosphorothioates and DNA bases in order to promote stability in cells. This could have affected their relative activities. Interestingly, while the guides were selected using hADAR2, they also performed well with the two hADAR1 isoforms. Another interesting observation from the *in cellula* data was the highly variable maximal editing between different guides, ranging from 23% to 57%. These levels did not correlate well with the EC_50_ measurements, which were overall quite similar (13–21 nM; Supplementary Figure S4). Similarly, variable steady-state editing was observed for the editing of the top guides *in vitro* (Figure 4) though in many cases the difference between these sequences was a single nucleotide.

The principal challenge to identifying optimized gRNAs for SDRE using selection-based approaches is the vast mutational space that must be covered. Given that there were ∼10^18^ possible sequences for our 31 nt gRNAs, and our *in vitro* editing assay contained in theory 6.7 × 10^10^ total molecules (based on the final RNA concentration in the 50 μl volume reaction), we clearly only screened a subset of the possibilities. In addition, the randomization scheme that was selected (91:3:3:3) imposed a bias on the portion of the mutational space that was interrogated (Supplementary Figure S7A), therefore some unique sequences were overrepresented in the initial ∼10^10^ total molecules pool. For example, in the initial pool, sequences with a single mismatch (besides the A/C mismatch opposite the target A) occupied 4.5% of the reads obtained by MiSeq. In fact, 52.7% of the reads for the initial pool had between 0 and 4 mismatches (Supplementary Figure S7A, B). Thus, each of the 86 unique sequences with a single mismatch found in the MiSeq data, were represented in average in 47 reads. On the other hand, the same sample contained in average only 1.2 reads for each of the 14,002 unique sequences found with five mismatches (Supplementary Figure S7B). At the outset of this work, we had no idea how many mismatches would prove to be optimal. In the final pool, after nine rounds of selection, the mode of the population was 8 mismatches (Supplementary Figure S7A), a number that was comparatively rare in the starting population. Furthermore, after 9 rounds of selection the average number of reads per unique sequence was the same for sequences with 3–12 mismatches (∼1.4, Supplementary Figure S7B), a redistribution that is not likely to occur from randomly selecting molecules from the initial pool. This shows that selection using ARMS PCR in fact worked. However, due to the biased distribution of the initial population, it did require many rounds of the assay before the top sequences started to appear. In fact, only after round 6 did the top sequences appear consistently among the top 10 most abundant sequences (Supplementary Figure S2), and even after nine rounds, these sequences were only present in 50–300 reads (out of 1 × 10^5^ analyzed reads). Moving forward, a higher initial degree of randomization could prove beneficial, allowing a more thorough coverage of the mutational space that contains a higher number of mismatched positions.

The development of this selection assay for efficient small gRNAs is an important step towards the successful application of SDRE in clinical settings. The observation that 8 out of the 10 guides selected from the 31 nt assay improved editing *in cellula* is a strong indication of the potential of the assay to help design small gRNAs. Nonetheless, there are a number of ways in which the assay itself might be modified for further improvement. For example, by using ADAR1 (p110 or p150) we might be able to select for gRNAs optimized for those isoforms. Other changes would be the use of different selective pressures (e.g. reaction time versus ADAR concentration), or the use of different randomization schemes. Finally, and most importantly, as gRNAs are selected for diverse targets, a more extensive dataset for the positions and identities of mismatches will emerge, perhaps resulting in generalized rules for gRNA design. With such rules, the mutational space at the outset of selection could be greatly reduced, or selection may not be required at all. Overall, methodologies and modifications that improve guide RNA design are key towards elevating SDRE to clinical applications.

## DATA AVAILABILITY

The scripts developed for the analysis of the MiSeq data of the selection assay, as well as the raw results are available in the Figshare repository https://figshare.com/articles/software/Selection-Assay/21997604.

## Supplementary Material

gkad098_Supplemental_File
