# Supplementary material for: Development of a selection assay for small guide RNAs that drive efficient site-directed RNA editing
Source: Nucleic Acids Res. 2023 Feb 25;51(7):e41. doi: 10.1093/nar/gkad098 (PMC10123091; doi:10.1093/nar/gkad098)
Supplement: gkad098_Supplemental_File [file gkad098_supplemental_file.pdf]

ARMS PCR validation

To select the edited products, we used a PCR based strategy called ARMS PCR (1, 2). This method is based on the observation that two- but not one- mismatches within the final three nucleotides of a PCR primer are required to disrupt amplification. To determine optimal ARMS primers, we started with fully edited and unedited control templates (i.e., one with a G at the editing site and the other with an A, Sup. Fig. 1A). We then chose forward primers whose 3' end matched the C in the antisense strand of the edited control but not the T in the unedited one. In addition to the mismatch at the last base position (position 0), we systematically tested two possible mismatches at the penultimate (-1) or the antepenultimate (-2) bases. These four primers were tested in combination with the P2 primer for their ability to selectively amplify the edited vs unedited controls. In addition, the Mg^{+2} concentration, as well as Taq and Vent(-exo) polymerases were explored (these two polymerases were selected because of their limited, or lack of proof-reading properties, respectively). Using qPCR (Sup. Fig. 1B), we determined that for the 21 nt substrate, the -1G forward primer (Sup. Table 1) in combination with Vent(exo-) polymerase, 18 cycles and a 2 mM Mg^{+2} final concentration, produced efficient amplification of the edited control but not the unedited control. Similarly, for the 31 nt substrate the -1G forward primer (Sup. Table 1), in combination with Vent(exo-) polymerase, 22 cycles and 2.5 mM Mg^{+2} final concentration were optimal (Sup. Fig. 1C).

Once we had determined the conditions for ARMS amplification on the control substrates, we tested whether we could enrich for edited molecules from a mix of the edited and unedited control substrates. Since the ARMS primer itself changes the target A to a G, we could not use this position as reference for the control's identity. Instead, two identifying mutations in the loop region of the unedited molecules (Sup. Fig. 1A) were introduced to distinguish between the two controls. When a 1:1 mixture was amplified by a Non-Selective PCR, a double A/G peak is visible in the target position, along with two double peaks (G/C and G/A) at the identifying mutations (Sup. Fig. 1D). In contrast, when the mixture was amplified using ARMS PCR, there were single peaks at the identifying mutations positions, both matching the G's in the edited control. Similar results were obtained with a 10:1 mix of unedited and edited control templates. These results confirm that the ARMS PCR effectively enriches for edited molecules.

REFERENCES

1. Medrano,R.F.V. and de Oliveira,C.A. (2014) Guidelines for the tetra-primer ARMS-PCR technique development. *Mol Biotechnol*, **56**, 599–608.
2. Little,S. (1995) Amplification-Refractory Mutation System (ARMS) Analysis of Point Mutations. *Curr Protoc Hum Genet*, **7**, 9.8.1–9.8.12.

ARMS Validation

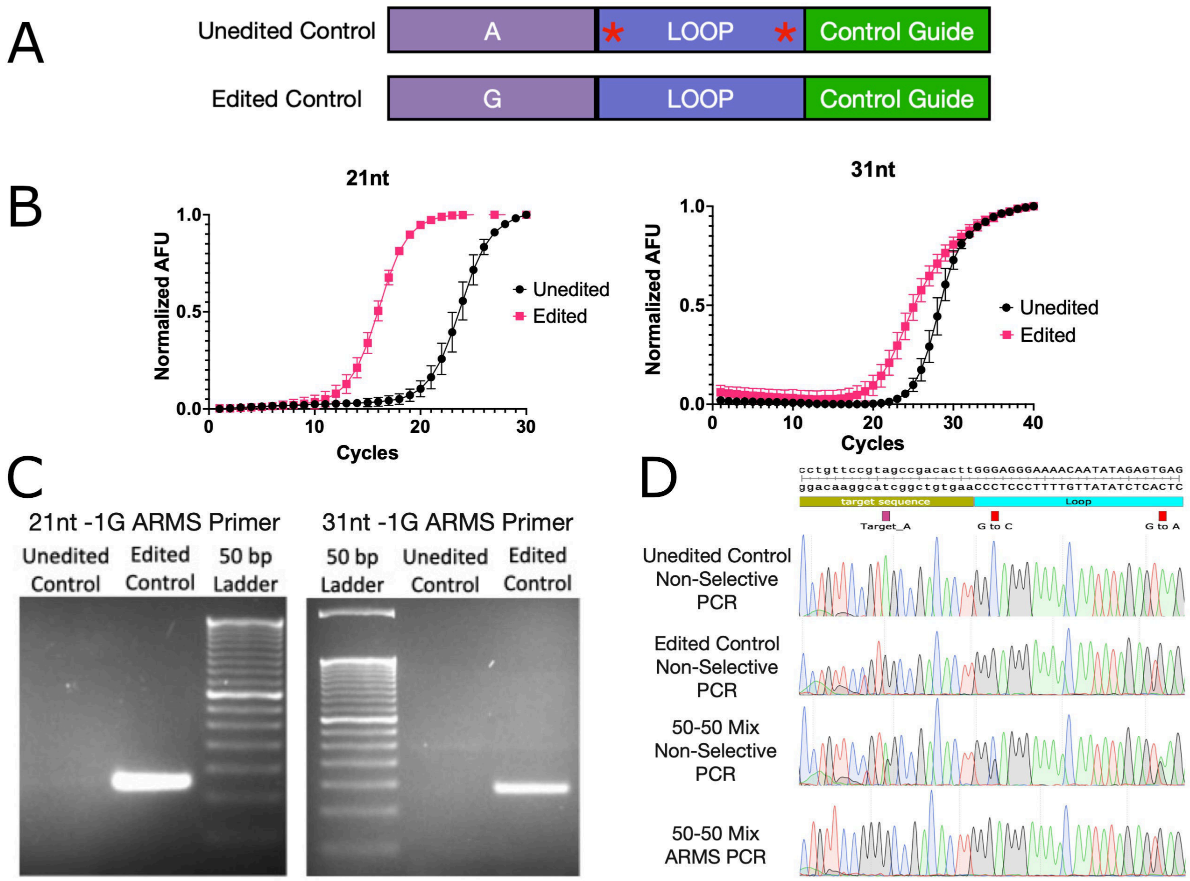

Supplemental Figure 1. ARMS PCR validation. A) Representation of the DNA substrates used to determine the conditions for ARMS PCR. Both controls contain a target region (purple), a loop region (violet) and a guide region (green) complementary to the target region. In the edited control, the target A was replaced with a G. Asterisks in the loop of the unedited control denote mutations in the loop region to differentiate the substrates during Sanger sequencing. **B)** qPCR curves used to determine the optimal number of cycles needed to amplify the edited control with minimal amplification of the unedited control. For comparison purposes, the fluorescence of the intercalating dye (dsGreen) was normalized to the highest value for each control. Error bars denote S.D. (n=3). **C)** Agarose gels showing the amplification of the edited control under the established conditions but not the unedited one. **D)** Example electropherograms for the 21 nt substrate showing that ARMS PCR can effectively amplify the edited control from mixtures of the edited and unedited controls. The sequence on top shows the location of the target A in the target region and the mutations in the Loop region in the unedited control.

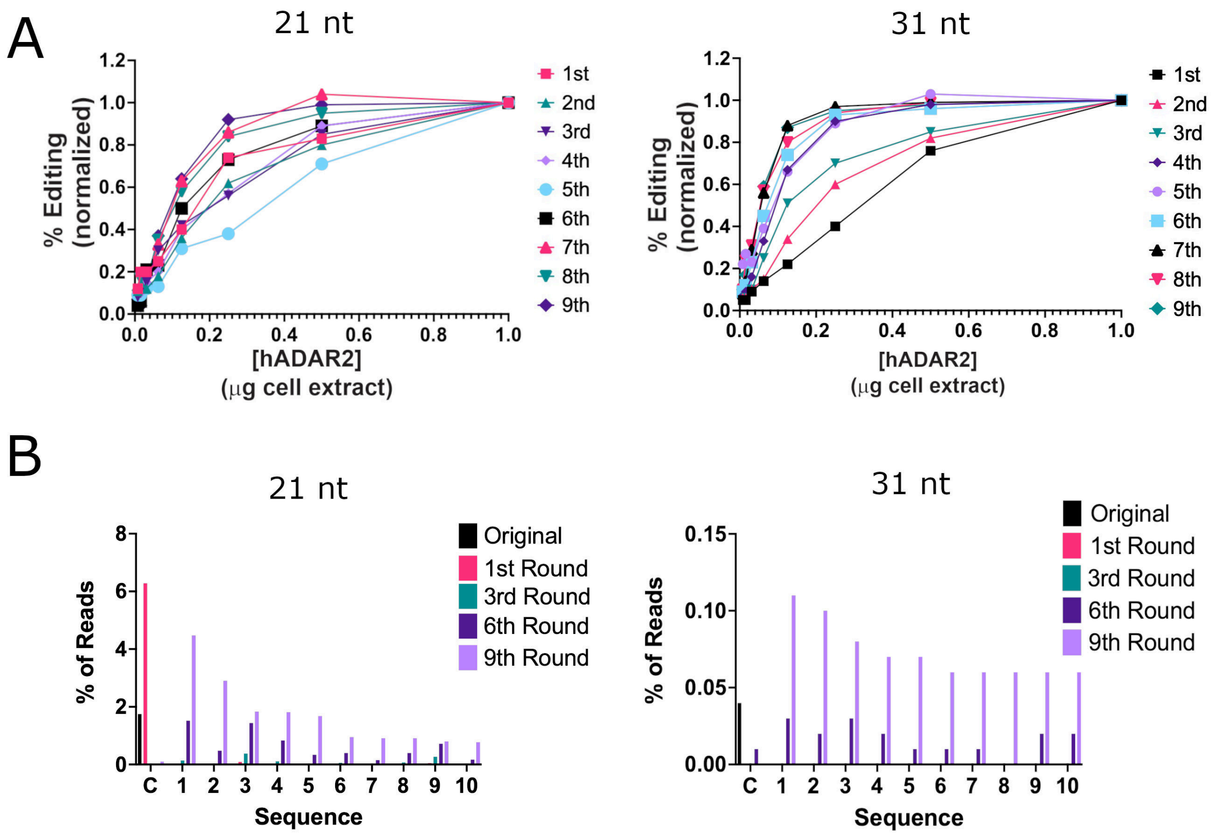

Supplemental Figure 2. Evaluation of the selection assay. A) Percent editing of the hairpin pool at different hADAR2 concentrations for all rounds of the assay. For comparison purposes, percent editing was normalized to the highest value, and hADAR2 concentrations were normalized to the amount of cell extract that produced the highest editing per substrate per round. n = 1 **B)** Enrichment rates of the top 10 most abundant hairpins and the control hairpin. The graphs show the percentage of reads in which each one of the hairpins was represented in the original substrate and in the regenerated substrate after 1,3, 6 and 9 rounds of selection assay.

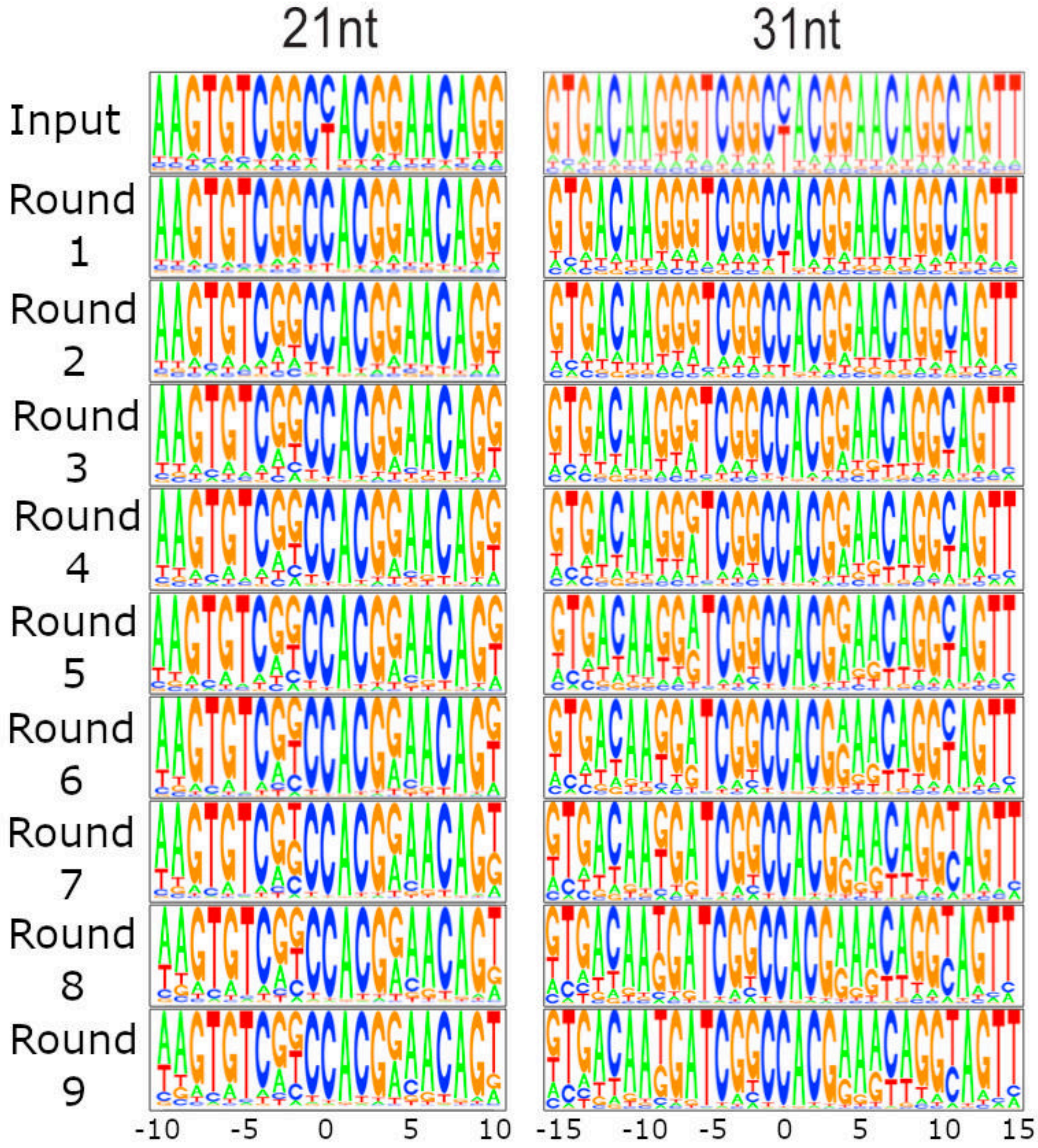

Supplemental Figure 3. Base preference during the assay. Logo plots of the original substrate and the regenerated substrates obtained after each round of the assay. Position 0 marks the position complementary to the target adenosine.

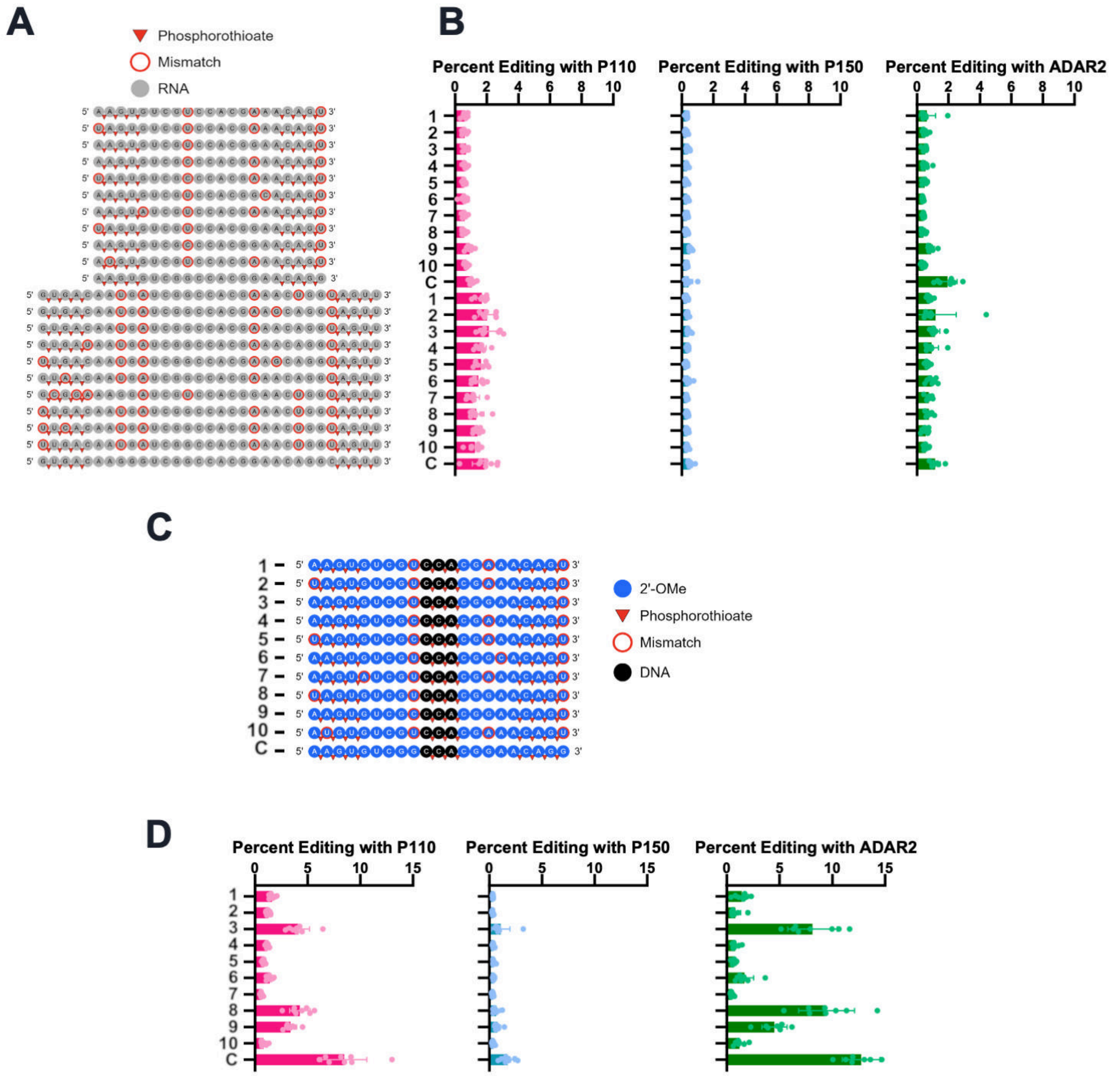

Supplemental Figure 4. Testing top guide RNAs *in cellula.* A) A map of the chemical modifications within the 21 and 31 nt ASOs of the 10 most abundant gRNAs as well as the control gRNA sequences. ASOs (100 nM) were transfected into HEK293T cells transiently expressing mCherry_P2A_eGFP W58X (UAG) and hADAR1-p110, hADAR1-p150 or hADAR2. **B)** Percent editing of the top 10 most abundant 21 and 31 nt gRNAs in comparison to the control. **C)** A map of the chemical modifications within the stabilized 21 nt ASOs of the 10 most abundant gRNAs as well as the control gRNA sequences. ASOs (100 nM) were directly transfected into HEK293T cells transiently expressing mCherry_P2A_eGFP W58X (UAG) and hADAR1-p110, hADAR1-p150 or hADAR2. **D)** Percent editing of the top 10 21 nt most abundant gRNAs in comparison to the control. Error bars represent S.D., and for all experiments, n=8.

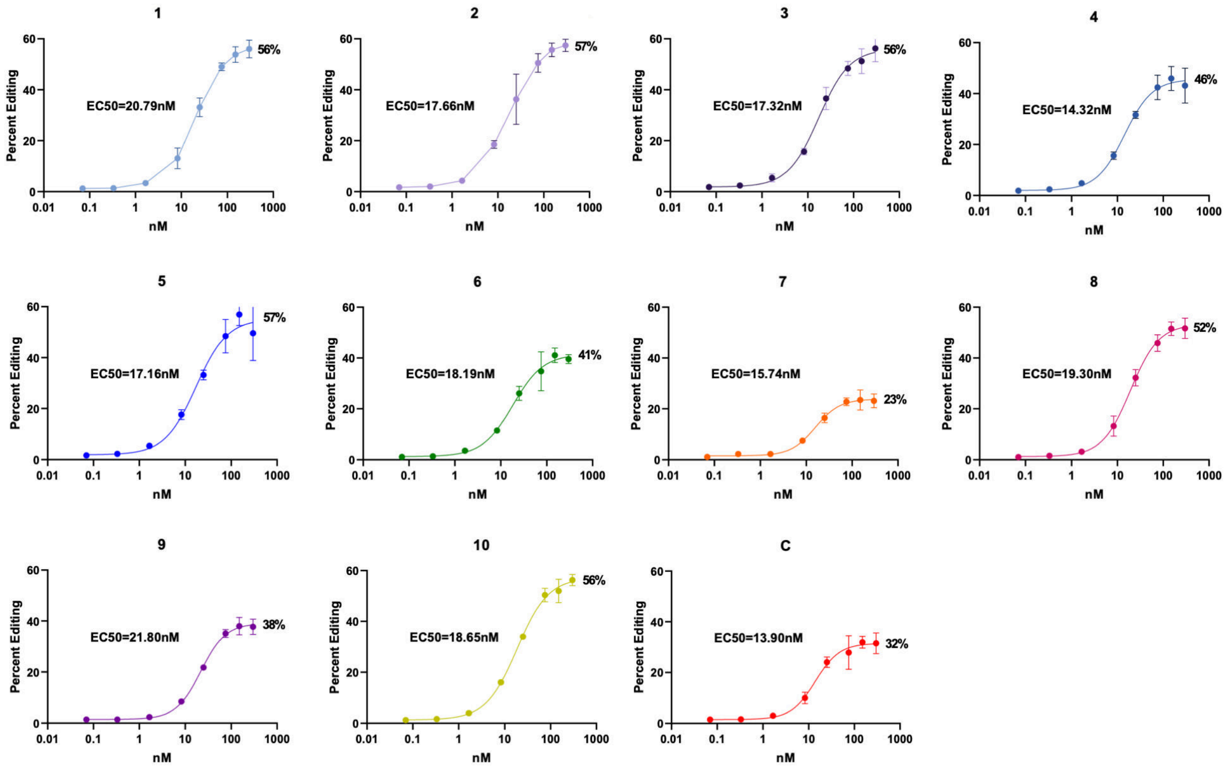

Supplemental Figure 5. Potency determination *in cellula*. Individual dose-response curves of the top 10 most abundant gRNAs and control gRNA (C) in HEK293T cells after co-transfection with plasmids encoding mCherry_P2A_eGFP W58X (UAG) and hADAR2. Each graph shows the maximum editing and the EC_{50} values. Error bars represent S.D., and for all data points, n=4.

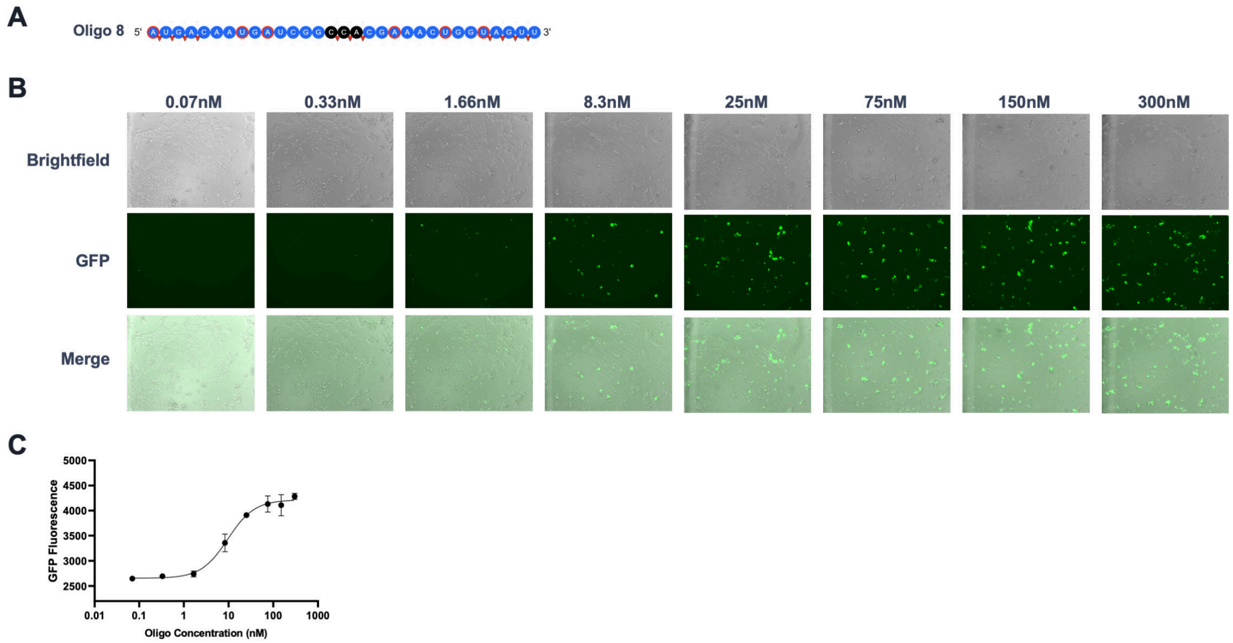

Supplemental Figure 6. Dose Response *in cellula.* A) A chemical modification map of the 31 nt gRNA #8. **B)** Representative images showing the increase in green fluorescence from the mCherry_P2A_eGFP W58X (UAG) reporter in HEK293T cells expressing hADAR2. A dose response was performed by transfecting gRNA #8 from (**A**) at eight different concentrations. **C)** The GFPsignal at different gRNA concentrations was plotted. Error bars represent S.D., and for all data points, n=4.

A

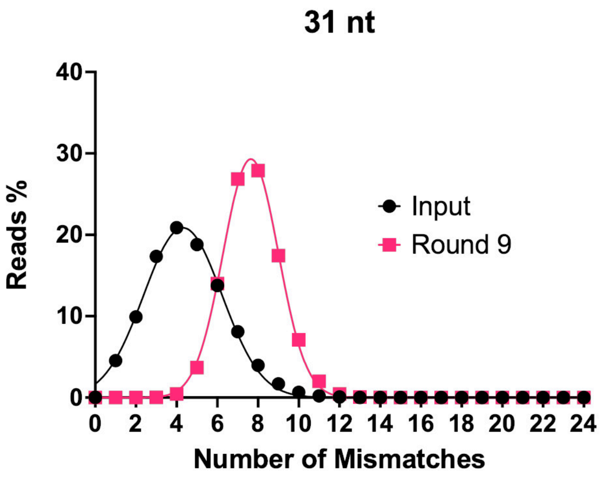

B

Input

Number of Mismatches	Number of Unique Sequence	Percentage	Reads/Unique Sequence (Average)
0	1	0.036	32.0
1	86	4.546	47.1
2	1683	9.916	5.2
3	6115	17.350	2.5
4	11378	20.872	1.6
5	14002	18.807	1.2
6	12635	13.766	1.0
7	9194	8.098	0.8
8	5403	3.968	0.7
9	2677	1.691	0.6
10	1115	0.641	0.5
11	438	0.205	0.4
12	145	0.079	0.5
13	48	0.016	0.3
14	9	0.007	0.7
15	4	0.001	0.3

Round 9

Number of Mismatches	Number of Unique Sequence	Percentage	Reads/Unique Sequence (Average)
0	0	0.000	0.0
1	0	0.000	0.0
2	0	0.000	0.0
3	19	0.020	1.1
4	347	0.450	1.4
5	2869	3.690	1.4
6	10878	14.000	1.4
7	20853	26.840	1.4
8	21667	27.880	1.4
9	13551	17.440	1.4
10	5514	7.100	1.4
11	1561	2.010	1.4
12	347	0.450	1.4
13	53	0.070	1.4
14	14	0.020	1.5
15	2	0.000	0.0

Supplemental Figure 7. Mismatch distribution within 31 nt hairpins. **A)** Histogram showing the percentage of MiSeq reads that contained 0 to 24 mismatches in the guide region in the input population, and after 9 rounds of selection. **B)** Summary of MiSeq data for the input population and after 9 rounds of selection showing the correspondence between number of mismatches, the number of unique sequences and the average number of reads per unique sequence.

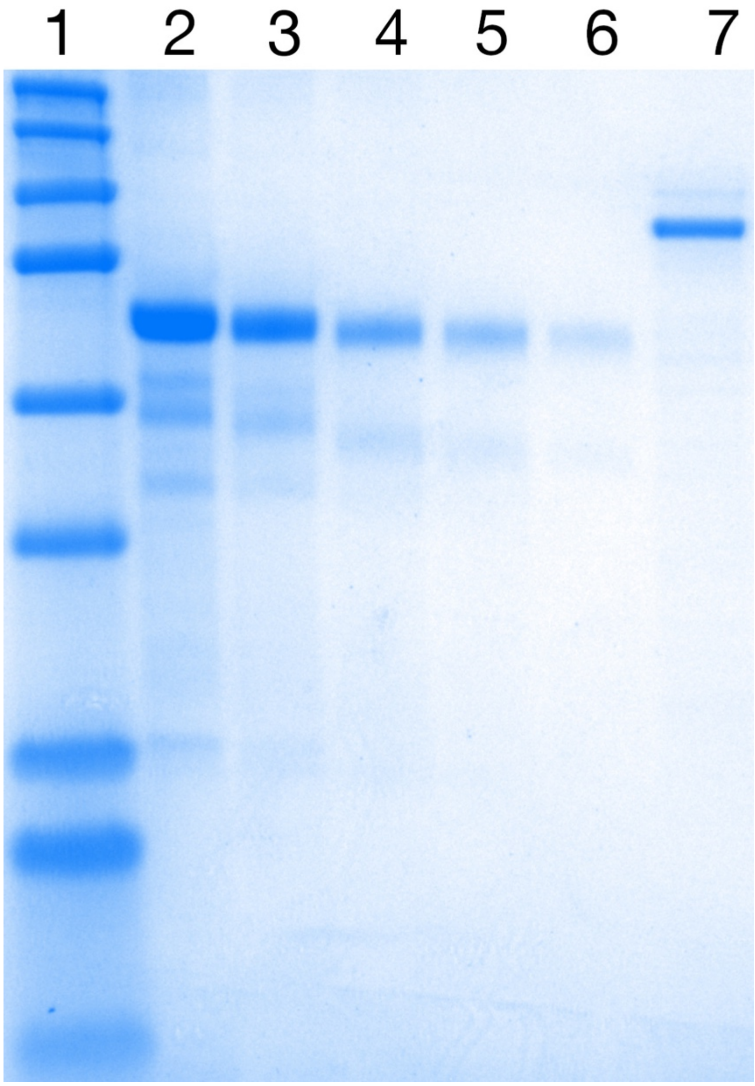

Supplemental Figure 8. hADAR2 purification and quantification*.* Polyacrylamide gel stained with GelCode Blue(Thermofisher) showing the obtained Purified hADAR2 (Lane 7) as well as the BSA standard serial dilution used for quantifying the protein obtained (Lanes 2 to 6). Lanes: 1-Molecular Marker (Kaleidoscope, BioRad), 2-BSA 500 ng/µl, 3-BSA 250 ng/µl, 4-BSA 125 ng/µl, 5-BSA 62.5 ng/µl, 6-BSA 31.25 ng/µl, 7-Purified hADAR2. The concentration of the purified protein was 228 ng/µl

Supplemental Table 1. Sequence of oligonucleotides used during the selection assay. N1, N2, N3, and N4 are the special hand mixes used to synthetize the random regions in the Guide Oligos, each one containing mainly the complementary nucleotide of the target oligo. For example, N1:03910303 is a hand mix that contains 3% A, 91%C, 3% G and 3% T. R represents a 50-50 T-C mix.

21nt Target Oligo	5'ACTCCGATGGCTGCAGTCCCTAATACGACTCACTATAGGGTGGTTGCCATAAGCGGATCATCGGGAGGAGAAACGGGCCTGTTCCGTAGCCGACACTTGGGAGGGAAAACAATATAGAGTGAG 3'
21nt Guide Oligo	5'TTTGGTTGGACTGGGTTTGGAACGCTCGCCTCGCTATC(N1:03910303)(N1)(N2:03030391)(N3:03039103)(N2)(N2)(N1)(N1)(N3)(N2)R(N3)(N1)(N1)(N3)(N4:91030303)(N1)(N4)(N1)(N2)(N2)CTCACTCTATATTGTTTTCCCTCCC 3'
31nt Target Oligo	5'ACTCCGATGGCTGCAGTCCCTAATACGACTCACTATAGGGTGGTTGCCATAAGCGGATCATCGGGAGGAGAAACGGGAACTGCCTGTTCCGTAGCCGACCCTTGTCACGGGAGGGAAAACAATATAGAGTGAG 3'
31nt Guide Oligo	5'TTTGGTTGGACTGGGTTTGGAACGCTCGCCTCGCTATC(N4:91030303)(N4)(N1:03910303)(N2:03030391)(N3:03039103)(N1)(N1)(N2)(N3)(N2)(N2)(N1)(N1)(N3)(N2)R(N3)(N1)(N1)(N3)(N4)(N1)(N1)(N1)(N2)(N2)(N3)(N2)(N1)(N4)(N1)CTCACTCTATATTGTTTTCCCTCCC 3'
Synthesis Primer fwd	5'ACTCCGATGGCTGCAGTCCCTAATACGACTCACTATAGGG 3'
P1	5'TGGTTGCCATAAGCGGATCATCGGGAGGAGAAACGGG 3'
P2	5'TTTGGTTGGACTGGGTTTGGAACGCTCGCCTCGCTATC 3'
21nt -1G ARMS fwd	5'CATCGGGAGGAGAAACGGGCCTGTTCCGGG 3'
31nt -1G ARMS fwd	5'GGAGGAGAAACGGGAACTGCCTGTTCCGGG 3'
21nt Regeneration Primer	5'GCCGACACTTGGGAGGGAAAACAATATAG 3'
31nt Regeneration Primer	5'ACGGGAGGGAAAACAATATAGAGTGAG 3'
MiSeq fwd	5'ACACTCTTTCCCTACACGACGCTCTTCCGATCTTGGTTGCCATAAGCGG 3'
MiSeq rev	5'GACTGGAGTTCAGACGTGTGCTCTTCCGATCTTTTGGTTGGACTGGGTTTGG 3'

Supplemental Table 2. Summary Statistics for MiSeq data analysis for the 21 nt hairpin after each round of the selection assay.

21nt						
	Original		1st Round		2nd Round	
Parameter	Total	Percentage	Total	Percentage	Total	Percentage
**Initial paired reads**	95749	100	101964	100	134712	100
**Reads with Indels**	7977	8.33	10264	10.07	19145	14.21
**Reads with mismatches**	2236	2.34	4398	4.31	4770	3.54
**Reads analyzed**	85536	89.33	87302	85.62	110797	82.25
**Different guide sequences**	46180	53.99	28599	32.76	53777	48.54
	3rd Round		4th Round		5th Round	
Parameter	Total	Percentage	Total	Percentage	Total	Percentage
**Initial paired reads**	119346	100	135791	100	96581	100
**Reads with Indels**	18985	15.91	24192	17.82	19732	20.43
**Reads with mismatches**	2577	2.16	2521	1.86	2303	2.38
**Reads analyzed**	97784	81.93	109078	80.33	74546	77.18
**Different guide sequences**	44474	45.48	47460	43.51	29569	39.67
	6th Round		7th Round		8th Round	
Parameter	Total	Percentage	Total	Percentage	Total	Percentage
**Initial paired reads**	103540	100	73026	100	44491	100
**Reads with Indels**	27685	26.74	20002	27.39	12810	28.79
**Reads with mismatches**	1532	1.48	2933	4.02	269	0.6
**Reads analyzed**	74323	71.78	50091	68.59	31412	70.6
**Different guide sequences**	24273	32.66	16653	33.25	10935	34.81
	9th Round					
Parameter	Total	Percentage				
**Initial paired reads**	116529	100				
**Reads with Indels**	32667	28.03				
**Reads with mismatches**	310	0.27				
**Reads analyzed**	83552	71.7				
**Different guide sequences**	18367	21.98				

Supplemental Table 3. Summary statistics for MiSeq data analysis for the 31 nt hairpin after each round of the selection assay.

31nt						
	Original		1st Round		2nd Round	
Parameter	Total	Percentage	Total	Percentage	Total	Percentage
**Initial paired reads**	109927	100	105158	100	99245	100
**Reads with Indels**	17011	15.47	13675	13	13003	13.1
**Reads with mismatches**	374	0.34	355	0.34	249	0.25
**Reads analyzed**	92542	84.18	91128	86.66	85993	86.65
**Different guide sequences**	67309	72.73	67401	73.96	64888	75.46
	3rd Round		4th Round		5th Round	
Parameter	Total	Percentage	Total	Percentage	Total	Percentage
**Initial paired reads**	136913	100	94470	100	45088	100
**Reads with Indels**	24637	17.99	14671	15.53	7407	16.43
**Reads with mismatches**	298	0.22	419	0.44	306	0.68
**Reads analyzed**	111978	81.79	79380	84.03	37375	82.89
**Different guide sequences**	94666	84.54	60716	76.49	34338	91.87
	6th Round		7th Round		8th Round	
Parameter	Total	Percentage	Total	Percentage	Total	Percentage
**Initial paired reads**	142950	100	78333	100	298284	100
**Reads with Indels**	21547	15.07	12252	15.64	45309	15.19
**Reads with mismatches**	313	0.22	326	0.42	745	0.25
**Reads analyzed**	121090	84.71	65755	83.94	252230	84.56
**Different guide sequences**	100175	82.73	55647	84.63	179580	71.2
	9th Round					
Parameter	Total	Percentage				
**Initial paired reads**	130887	100				
**Reads with Indels**	22651	17.31				
**Reads with mismatches**	387	0.3				
**Reads analyzed**	107849	82.4				
**Different guide sequences**	77706	72.05				
